# Pharmacological Potential of Sea Cucumbers

**DOI:** 10.3390/ijms19051342

**Published:** 2018-05-02

**Authors:** Yuri Khotimchenko

**Affiliations:** 1 School of Biomedicine, Far Eastern Federal University, 8 ul. Sukhanova, Vladivostok 690950, Russia; yukhotimchenko@mail.ru; Tel.: +7-914-710-5906; 2 National Scientific Center for Marine Biology, Far Eastern Branch of Russian Academy of Sciences, Vladivostok 690041, Russia

**Keywords:** Echinodermata, Holothuroidea, sea cucumber, polysaccharides, fucosylated chondroitin sulfate, glycans, saponins, triterpene glycosides, ceramides

## Abstract

This review presents a detailed analysis of published research data focused on the pharmacological activity exerted by biologically active compounds isolated from sea cucumbers belonging to the class of Holothuroidea, phylum Echinodermata. The review contains descriptions of the structure, physico-chemical properties and pharmacological effects of these active substances. Particular attention is given to compounds with anticoagulant, antithrombotic, antioxidant, anticancer, anti-infectious, immune-stimulating and anti-ACE (angiotensin converting enzyme) activities as well as to the substances exerting a regulating influence on lipid and carbohydrate metabolism. All these compounds may be considered as prototypes for development of new pharmaceutical substances and medicines.

## 1. Introduction

Worldwide marine pharmacy confirms the enormous potential of sea species as a source for development of novel pharmaceutical substances and medicines. The phylogenetic (or macrotaxonomical) diversity in marine and fresh waters is much higher than that on land. Out of 33 phyla of multicellular organisms 31 phyla are found in sea waters, 17 phyla in fresh waters and only 11 phyla on land [1]. Due to numerous adaptations to different environmental factors many marine animals and plants have abilities to synthesize unique secondary metabolites, which are not typical of terrestrial species. Many of these compounds possess pronounced pharmacological activity. Sources for novel pharmacologically active compounds of marine origin are bacteria (Eubacteria), cyanobacteria and protists, several phyla of invertebrates such as sea sponges (Porifera), bryozoans (Bryozoa), mollusks (Mollusca), echinoderms (Echinodermata) and tunicates of the subphylum Tunicata. Many researchers consider marine invertebrates, such as sponges, much more fruitful sources of novel antitumor, antiviral, and anti-inflammatory agents than any group of terrestrial species [2].

Since the late 1960s, directed chemical synthesis in drug discovery and development has been considered the most preferable approach. Since the beginning of the new millennium, methods of drug discovery have changed. Nowadays, natural compounds from various species are the main focus for pharmacological studies due to achievements in new biomedical technologies such as high throughput screening and development of sensitive cell and molecular targets for drug candidates.

Currently, approximately 27,000 different chemical compounds have been isolated from marine species [3], and many of them exert pharmacological effects. The compounds vary from simple linear peptides such as dolastatins, to complex macrocyclic polyethers such as halichondrin B. Biologically active compounds of marine origin may be used as pharmaceutical substances as well as initial prototypes or synthones for development of compounds with novel or improved pharmacological characteristics.

The present review is devoted to the pharmacological potential of a very promising group of marine invertebrates, the sea cucumbers. They have gained a lot of attention from researchers worldwide within recent decades because of their nutritive values [4] as well as their beneficial influence on human health and possible therapeutic uses. The spectrum of active compounds chemically identified from these animals is quite diverse and includes polysaccharides such as glycosaminoglycans (mucopolysacharides) including neutral glycans, fucosylated chondroitin sulfates and sulfated fucans, peptides, phospholipids and glycolipids, including glycosphingolipids (cerebrosides), polyunsaturated fatty acids, phenols, and triterpene glycosides (saponins) [5,6,7,8,9,10,11,12]. In this review, the main attention is given to the compounds for which pharmacological activity has been scientifically demonstrated, physico-chemical properties required for their further standardization have been described and their mechanisms of action are more or less clear (Table 1).

## 2. Biologically Active Compounds with Anticoagulant and Antithrombotic Activity

The first studies estimating anticoagulant activity of biologically active compounds from sea cucumbers were carried out and published in the 1980s when large amounts of sulfated polysaccharides rich in fucose were found in the body wall of the sea cucumber *Holothuria grisea* (*Ludwigothurea grisea*) [13]. It contained several fractions of this polysaccharide varying in molecular weight and chemical structure. The high molecular weight fraction contained the most fucose and small amounts of galactose and aminosugars. The polysaccharide fraction with lower molecular weight generally consisted of sulfated fucans. The fraction with the lowest molecular weight making up the largest portion of the polysaccharide contents of the sea cucumber was composed of sulfated polysaccharides with approximately equimolar quantities of glucuronic acid, *N*-acetylgalactosamine, and fucose. Also, it had higher sulfate content then the two higher molecular weight fractions mentioned above [14].

**Figure 1:**
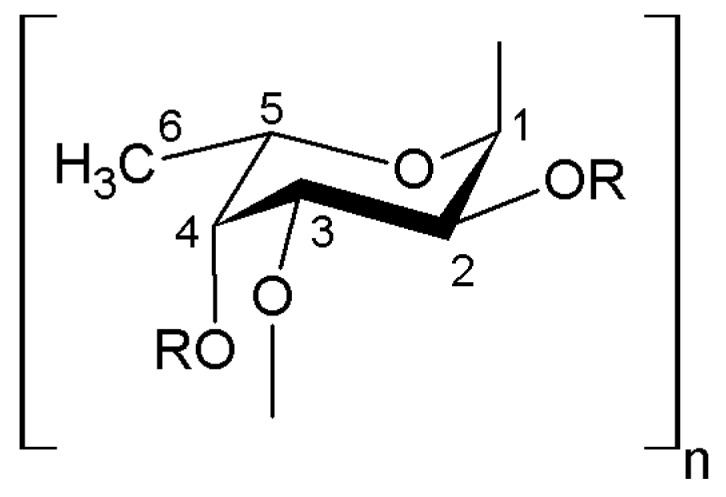
The basic backbone structure of 1-3-linked fucose residues in many sulfated fucans from marine organisms. *R* may be hydrogen, sulfate, or a galactose or fucose side chain.

**Figure 2:**
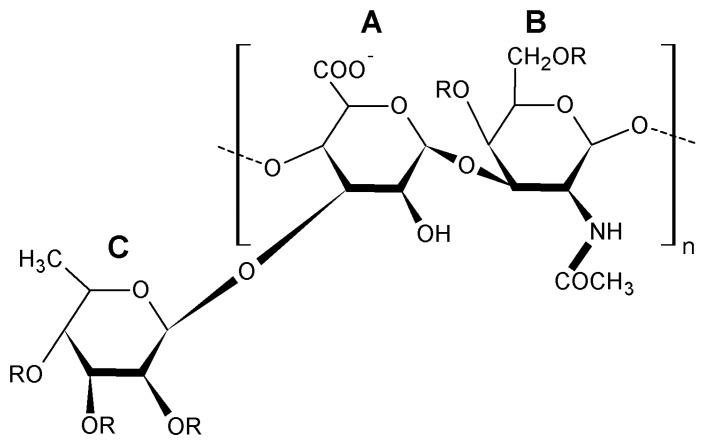
Preponderant structure of the fucosylated chondroitin sulfate. The backbone is made up by repeating disaccharide units of alternating β-d-glucuronic acid (A) and *N*-acetyl-β-d-glucosamine (B). The β-d-glucuronic acid residues bear 2,4-disulfated fucose branches (C) at the 3-*O*-position. R may be hydrogen or sulfate.

**Figure 3:**
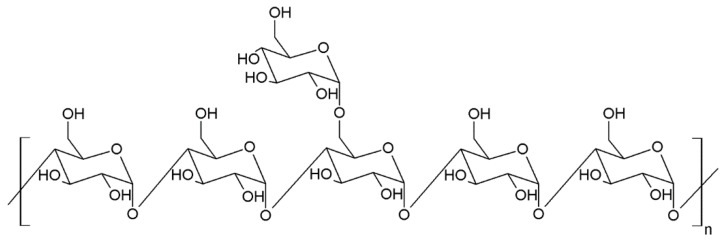
Neutral glucan from sea cucumber *Holothuria edulis*. The major component of polysaccharide is an α-(1→4)-d-glucan branched with a single α-d-glucose at C-6 every five residues on average.

Nowadays, at least three types of polysaccharides are known to be in the body wall of sea cucumbers. They are sulfated fucans, fucosylated chondroitin sulfates and neutral glycans [15,16,17] (Figure 1, Figure 2 and Figure 3). The first two of them were identified in all species that were investigated whereas neutral α-glucan was isolated only from the sea cucumber *Holothuria edulis*.

**Figure 4:**
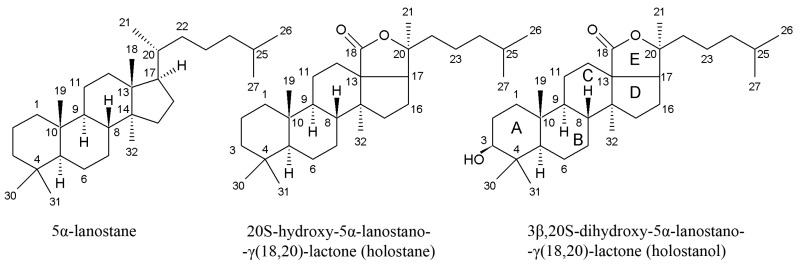
Structures of lanostane, holostane and holostanol. A, B, C, D, E: rings of the pentacyclic triterpene.

All these polysaccharides consist of similar structural features of repeating units of oligosaccharide, whose residues have specific patterns of sulfation. All echinoderms contain linear polymer sulfated fucans consisting of regular tandem repeats such as di-, tri- or tetrasaccharide repeating units with defined glycosidic linkages and distinctive sulfation patterns at O-2 and O-4 [15]. Each holothurian species contains polysaccharides with specific sulfation patterns and positioning of the glycosidic linkage. For example, tissues of *H. grisea* contain sulfated fucan with the following sequences of residues: [3-α-l-Fucp-2,4(OSO3)-1→3-α-l-Fucp-1→3-α-l-Fucp-2(OSO3)-1→3-α-l-Fucp-2(OSO3)-1]n (Figure 4) [18,19].

The sea cucumber *Isostichopus badionotus* was found to have the repeating tetrasaccharide unit [→3Fuc(2S,4S)α1→3Fuc(2S)α1→3Fuc(2S)α1→3Fucα1→]n [20]. *Apostichopus japonicus* (*Stichopus japonicas*) was shown to have two different types of sulfated fucan polymers. One of them consists of (1→3)-linked linear fucosyl residues that are substituted at C-4 with fucosyl residues and has 3.41 mmol fucose/g and 2.35 mmol sulfate/g. Using gel permeation chromatography, its molecular weight was found to be 9 kDa. The second one is generally a chain of unbranched (1→3)-linked fucosyl residues. It has 3.90 mmol fucose/g and 3.07 mmol sulfate/g contents, and its molecular weight is 32 kDa. Both have unbranched (1→3)-linked fucosyl residues [21].

Comparison of chemical composition and properties of sulfated fucans from the body wall of these sea cucumbers is given in the Table 2. It is easily noted that sulfated fucans from *Apostichopus japonicas* (*Stichopus japonicus*) and *Holothuria nobilis* have substantially high values of M_W_/Mn serving as indicators of the width of molecular weight distribution or, in other words, homogeneity and dispersibility of the polysaccharide.

Glycosaminoglycans from the body wall of holothurian species are characterized by a chondroitin sulfate back-bone having a large number of sulfated fucose branches. Thus, they are sometimes termed fucosylated chondroitin sulfates (FuCS). As a natural substance, FuCS is a specific class not found in any species except the echinoderm sea cucumbers and considered a unique sulfated glycosaminoglycan with different structure and functions in comparison to known mammalian glycosaminoglycans such as dermatan sulfate, keratansulfate, heparin, and chondroitin sulfate [22]. Structurally, holothurian glycosaminoglycan is composed of common polysaccharides found in mammalian chondroitin sulfates but their uronic acid residues bear unusual sulfated fucosyl branches O-linked to С-3 carbon [23]. Sulfated fucose branches in these glycosaminoglycans are linked to the O-3 position of β-d-glucuronate and O-4 and/or O-6 positions of *N*-acetyl-β-d-galactosamine [24,25]. FuCS found in the walls of the sea cucumber *Apostichopus japonicus* from the Qingdao Sea of China consists of *N*-acetyl-β-d-galactosamine (GalNAc), β-d-glucuronic acid (GlcUA), α-l-fucose (Fuc) and sulfate ester with approximately the molecular ratio of 1:0.97:1.13:3.85, respectively, composing the backbone (→4)GlcUAβ(1→3)GalNAcβ(1→) [26]. Branches of sulfated fucose are generally composed of fucopyranosyl residues linked to O-3 of β-d-glucuronic acid and O-6 and O-4 positions of the *N*-acetylgalactosamine moiety in 46.5%, 8.7% and 43.9% cases, respectively. Results of several studies authored by Vieira et al. have demonstrated that glycan from *H. grisea* has a core similar to mammalian chondroitin sulfate but is substituted with fucose-2,4 disulfated branches at the 3-position of the glucuronic acid residues [14,27]. The chemical composition and structure of the glycosaminoglycans from *A. japonicus* and *H. grisea* are quite different. The molar ratio of these components in glycosaminoglycan from *A. japonicus* was 1:0.99:0.94:3.73 [28] whereas this ratio of glycosaminoglycan from *H. grisea* was 0.92:1:1.23:0.7 [23]. Table 2 gives parameters of the chemical structure and physicochemical properties of glycosaminoglycans found in the body wall of four sea cucumbers. It is interesting that M_W_/Mn parameter of glycosaminoglycans isolated from these three sea cucumber species is quite close to that of heparin isolated from porcine intestine.

Two FuCS polysaccharides were found in the sea cucumber *Eupentacta fraudatrix*. The core pattern of one polymer consisted of chondroitin sulfate A and E units in a ratio of about 1:1. Besides the chondroitin sulfate A and E fragments, the backbone of the second polymer contained unusual disaccharide repeating units →4)-β-d-GlcpA2S3S-(1→3)-β-d-GalpNAc6S-(1→. The main pattern of branched chains of both polymers was a α-l-Fucp3S4S unit linked to O-3 of GlcA residues. Also, there was another type of branching found in the disaccharide fragment α-l-Fucp-(1→2)-α-l-Fucp3S4S-(1→ linked to O-3 of GlcA [29]. It is very likely to be the first isolation and characterization describing the presence of more than one structurally different FuCS in one species of sea cucumber. The backbone of FuCS isolated from the sea cucumber *Cucumaria frondosa* was found to consist of fragments of chondroitin sulfate A and E with the rather uncommon disaccharide repeating units →3)-β-d-GalNAc4S6S-(1→4)-β-d-GlcA3S-(1→ and →3)-β-d-GalNAc4S-(1→4)-β-d-GlcA3S-(1→. This polysaccharide was discovered to possess three types of branches. Two of these branches were determined as α-l-Fuc p3S4S and α-l-Fuc p2S4S linked to O-3 of Glc pA residues, while the third chain was per-*O*-sulfated α-l-Fuc attached to O-6 of Gal pNAc residue. The ratio of these branches was 5:2:1 [30]. Sea cucumber *Cucumaria frondosa* contains a more complicated FuCS than the FuCS isolated from the abovementioned holothurian species [31]. It seems that the glycosaminoglycan structure(s) in the same species grown in different geographic areas is/are different. FuCS from the sea cucumber *Massinium magnum* has a backbone generally consisting of chondroitin sulfate E units with a small portion (about 10%) of chondroitin sulfate A. Virtually one type of branch Fuc3S4S attached to O-3 of GlcA residues was found in the polysaccharide molecules. The main repeating blocks are presented with (→4)-[α-l-Fuc3S4S-(1→3)]-β-d-GlcA-(1→3)-β-d-GalNAc4S6S-(1→), whereas the minor repeating units were found to be (→4)-[α-l-Fuc3S4S-(1→3)]-β-d-GlcA-(1→3)-β-d-GalNAc4S-(1→) [32].

The sea cucumber *Holothuria mexicana* contains another structurally different FuCS. Its core backbone had chondroitin O-6 sulfate, while the major O-4 sulfated branches were linked to the O-3 position of glucuronic acid in almost every disaccharide unit [33].

Sulfated fucans and FuCS possess pharmacological activity exerting heparin-like anticoagulant [34,35,36,37] and antithrombotic effects [34,38] whereas neutral glucans are inactive. Anticoagulant effects of polysaccharides from sea cucumbers were demonstrated using activated partial thromboplastin time (APTT), prothrombin time (PT) and thrombin time (TT) and compared with unfractionated heparin from mammalian sources. APTT, PT, and TT measurements are commonly used to assess the ability to inhibit intrinsic, extrinsic and common pathways of the coagulation cascade, respectively, reducing blood clotting reactions [17,39] (Table 3). Under in vitro conditions, antithrombotic activity can be estimated by measuring the length and weight of the thrombus generated in an artificial blood vessel [20] and also assessing either inhibition of thrombin by heparin cofactor II and antithrombin or factor Xa activation by antithrombin. (Table 4).

The results of comparative studies suggest that anticoagulant activity of the holothurian polysaccharides generally depends on their molecular weight, presence of monosaccharide units, and amount and position of sulfate groups [23,24,40,41,42,43,44,45]. It was confirmed by Wu et al. [44], that anticoagulant activity of the FuCS isolated from the sea cucumber *Thelenota ananas* measured by APTT assays varies by its molecular weight in a logarithmic-like mode. Similarly, antithrombotic activity of FuCS from *Holothuria forskali* dependeds on the polysaccharide molecular weight [40]. In vitro comparative studies of anticoagulant and antithrombotic activities of sulfated fucan and FuCS from the sea cucumber *Isostichopus badionotus* showed that these two polymers exert very different effects. Sulfated fucan activity is characterized by significantly lower anticoagulant activity and moderate antithrombotic influence compared to the FuCS. Mechanisms for these effects were found to be different as well. Sulfated fucan potentiates antithrombin activities of factors IIa and Xa. FuCS mostly activates heparin cofactor II (HC II) up-regulating factor IIa and factor Xa. The effects of these polysaccharides were very similar to those of fucan and FuCS from sea cucumber *H. grisea* despite the fact that some structural patterns of the sulfated polysaccharides were different [46]. The authors of this study concluded that sulfated fucan isolated from the sea cucumber *Isostichopus badionotus* may be considered as a good prototype for a promising antithrombotic medicine with very low risk of undesirable bleeding [20].

Glycosaminoglycan from *A. japonicus* showed signs of anticoagulant activity as APTT and TT were significantly longer although in PT assays, clotting inhibition was not registered. According to APTT activity, the efficacy of this glycosaminoglycan was close to standard unfractionated heparin. Anticoagulant activity of heparin in concentrations of 170 μg/mL and lower was just the same as that of this natural glycosaminoglycan. An increase in concentration resulted in lowering anticoagulant activity of the polysaccharides whereas heparin activity continued to rise. Anticoagulant activity of glycosaminoglycans strictly depends on the amount of sulfated fucose branches [47,48]. These results were confirmed in studies devoted to estimating APTT using intact glycosaminoglycan and defucosylated glycosaminoglycan at 250 μg/mL. APTT after exposure to these polysaccharides was 244.50 and 44.24, respectively. TT activity of glycosaminoglycan was slightly higher than that of heparin at the same concentration. Such results suggest that glycosaminoglycan from *A. japonicus* can inhibit both the intrinsic and common pathways of coagulation and thrombin activity as well as conversion of fibrinogen into fibrin [5]. At the same time, FuCS from the sea cucumber *Massinium magnum* possesses lower anticoagulant activity than heparin but higher than enoxaparin, and does not provoke platelet aggregation, even in the platelet-enriched plasma [32].

Glycosaminoglycans from sea cucumbers are known to exert anticoagulant activity through numerous mechanisms. The most important mechanisms are activation of heparin cofactor II (HCII) resulting in thrombin inhibition with consecutive inactivation of factor VII and prevention of factor X activation via intrinsic tenase complex [49]. Since the activity of glycosaminoglycans isolated from sea cucumbers does not depend on the antithrombin concentration, the risk of induced bleeding with their use is lower than that of heparin [50].

Although the majority of fucosylated glycosaminoglycans have high anticoagulant and antithrombotic activities [37,51], a lot of them exert some side effects such as induction of factor XII activity and platelet aggregation [51].

Therefore, from the pharmacological point of view, research studies focusing on preparations of low molecular polysaccharides and investigating their pharmacological effects are more promising. Polysaccharide derivatives with low molecular weight may not have the adverse side effects typical of high molecular polymers. On the other hand, the behavior of high molecular weight polysaccharides may be more controllable.

It has been shown that induction of thrombin inhibition as well as ongoing heparin-dependent APTT activity of the oligosaccharide chain consisting of 16–18 units must react with coagulation factors [52]. In the study of Yang et al. [26] oligosaccharides were prepared using copper-induced controlled free-radical depolymerization of intact FuCS isolated from the sea cucumber *Apostichopus japonicus* (M_W_ of 98,070). There were three fractions of oligosaccharides obtained with molecular weights 41,119, 24,755, and 8871 Da. Analysis of monosaccharide and sulfation contents showed all three fractions were structurally very close to each other. In addition, cellulose acetate membrane electrophoresis confirmed that generally, the main structural patterns of natural high molecular and low molecular glycosaminoglycans were not different. That means the depolymerization process does not affect the core structure of these biopolymers.

Prolongation of APTT activity was not a result of a specific *N*-acetylaminoglycan from *Aposticopus japonicus* consisting of at least 4–6 units [26]. That study confirmed that such low molecular weight glycosaminoglycan from sea cucumber does not increase the risk of bleeding that is typical of heparin as well as low molecular weight heparins. Comparison studies of anticoagulant activity exerted by glycosaminoglycan fragments with molecular weight 24,755 Da in various concentrations and low molecular weight heparin (LMWH, M_W_ = 3500 Da) in the same concentrations were carried out under in vitro conditions. The results showed that APTT activity of depolymerized glycosaminoglycan and LMWH are linearly dependent on the concentration. Moreover, activity of both of these compounds at equivalent therapeutic doses was found to be almost the same but depolymerized glycosaminoglycan demonstrated significantly lower risk of bleeding [26]. It should be noted that the lower the molecular weight of depolymerized glycosaminoglycans is, the lower their anticoagulant activity. The difference between an anticoagulant and a hemorrhagic dose of the low molecular glycosaminoglycan was significantly larger than that of natural biopolymers suggesting that depolymerized compounds exert antithrombotic effects with lower risk of hemorrhagic complications [24]. These results were confirmed in in vivo experiments using an electrically induced arterial thrombosis model in rats. Anticoagulant and antithrombotic activities of FuCS isolated from the sea cucumber *Cucumaria frondosa* were more promising because the difference between antithrombotic and hemorrhagic doses was much wider than that of natural polysaccharides [53]. The sulfation pattern of FuCS was shown to slightly affect anticoagulation and antithrombosis in contrast to their molecular weight. Fractions of FuCS with low molecular weight depolarized using hydrogen peroxide also have shown a lower risk of bleeding [28]. This depolymerized glycosaminoglycan was shown to exert its activity through two different mechanisms. The first one is related to the heparin cofactor II (HCII)-dependent inhibition of thrombin. The second mechanism involves antithrombin III (AT III)- and HCII-independent inhibition of the factor X activation by the factor IXa-factor VIIIa complex [49]. Other researchers suggest that there are at least two anticoagulant mechanisms typical of the FuCS i.e., thrombin inhibition by heparin cofactor II, and inhibition of factor-Xa and thrombin generation by the tenase and pro-thrombinase complexes [35,37]. Degraded FuCS obtained from the natural polymer isolated from *Holothuria mexicana* was shown to have anticoagulant activity depending on its molecular weight with low bleeding risk in comparison to low molecular weight heparin [33].

Numerous recent studies have shown that oligosaccharides with low molecular weight exert a more promising antithrombotic–hemorrhagic ratio in comparison to unfractionated heparin (UFH) and LMWH [43,50]. Experimental results confirmed that a series of depolymerized fucosylated glycosaminoglycans with various molecular weights obtained through deaminative cleavage of glycosaminoglycans isolated from the sea cucumber *Thelenota ananas* dramatically increased human plasma APTT. It was determined that APTT was doubled even if the concentrations of depolymerized fucosylated glycosaminoglycans with molecular weight higher than 6000 Da were lower than 12 μg/mL suggesting that these polysaccharides decelerate intrinsic coagulation. This mechanism was confirmed by observations that depolymerized fucosylated glycosaminoglycans added in the concentration range of 0.005–2.560 mg/mL prolonged APTT in a concentration-dependent manner. Moreover, the higher the molecular weight of the depolymerized fucosylated glycosaminoglycans, the more APTT was prolonged in a logarithmical scale [54]. These results correspond to the findings of some previous studies [43,55]. Thus, depolymerized fucosylated glycosaminoglycans can be considered as promising anticoagulant drug prototypes only if their molecular weight is higher than 6000 Da [43].

Depolymerized fucosylated glycosaminoglycans were shown to dramatically inhibit anti-factor Xase (tenase-mediated factor X) with the half maximal effective concentration (EC_50_) of approximately 20–40 ng/mL [54].

Heparin cofactor II working as a catalyst, enhanced thrombin inhibiting activity of depolymerized fucosylated glycosaminoglycans with EC_50_ < 286 ng/mL. These polysaccharides also demonstrated anti-factor Xase and anti-IIa activity working as free-radical depolymerization products. As mentioned before, deaminative cleavage-prepared depolymerized fucosylated glycosaminoglycans with molecular weight 6000 Da and higher prolong APTT and inhibit thrombin activity and Xa factor synthesis. Their mode of action is very likely to be an affinity for HCII and the intrinsic tenase complex. These findings suggest that the role of molecular weight on inhibition of factor Xase and HCII-mediated thrombin is very slight [54].

FuCS is known to induce factor XII and platelet activity depending on its molecular weight. That is why development of FuCS depolymerization for synthesis of low molecular fragments with selective inhibition of intrinsic tenase seems reasonable. Depolymerized oligosaccharides obtained by this method for β-eliminative depolymerization had molecular weight between 3200 and 8800 Da and showed potent anticoagulant activities because they inhibited intrinsic tenase and maybe diminished or stopped factor XII or platelets. The lowering of their molecular weight resulted in diminished or even eliminated anticoagulant activity [5].

The results of the articles mentioned in this section suggest that the most promising substances isolated from sea cucumbers with anticoagulant and antiplatelet activity for drug prototypes are the low molecular fucosylated glycosaminoglycan derivatives. Currently, depolymerized holothurian glycosaminoglycans are in clinical trials in Japan focusing on creating a new antiplatelet medicine [26].

Two different oral forms of FuCS were tested experimentally [38]. It was found that acid-resistant tablets provide more pronounced and longer anticoagulant effects than an aqueous solution and had no influence on bleeding tendency and arterial blood pressure. Furthermore, preclinical studies of animals with arterial thrombosis induced by endothelial injury showed that coated tablets with FuCS exerted strong anticoagulant effects whereas the aqueous solution of this polysaccharide had no effect. The same studies used several common anticoagulants as positive controls and they were shown to dramatically increase bleeding tendencies [38]. The author concludes that FuCS from the sea cucumbers may be considered a promising source for a new oral anticoagulant drug.

## 3. Biologically Active Compounds with Anticancer Activity

### 3.1. Triterpene Glycosides

The first study suggesting anticancer properties of biologically active compounds found in sea cucumbers was the article authored by Nigelli in 1952 [56] demonstrating that an injection of a glycoside fraction from sea cucumber *Actinopyga agassizi* consisting of a number of triterpene glycosides (holothurins) into a sarcoma-180 node inhibited tumor growth in mice. Later, it was shown that healthy mice injected with Krebs-2 ascitic tumor cells and treated with holothurin prevented induction of the tumor growth [57]. Holothurin was also shown to inhibit growth of epidermal carcinoma cells [58].

**Figure 5:**
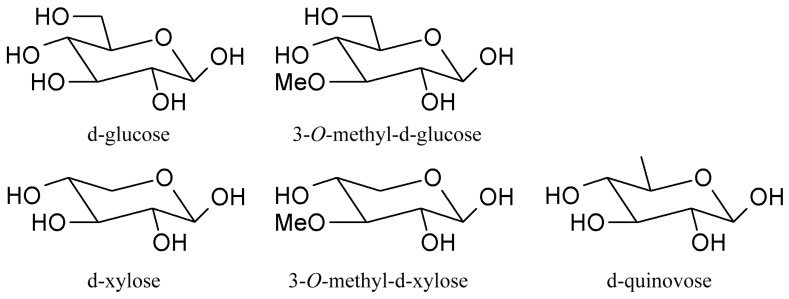
Common sugar units present in sea cucumber triterpene glycosides [59].

**Figure 6:**
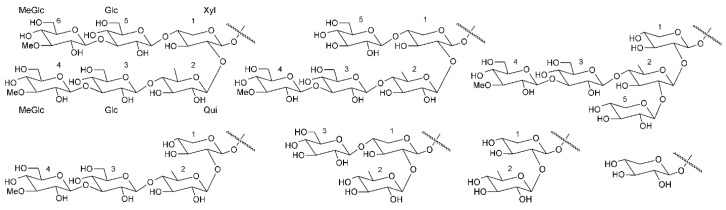
Some common carbohydrate architectures in the sea cucumber triterpene glycosides. The sugar moieties are 3-*O*-methyl-d-glucose (MeGlc), d-glucose (Glc), d-xylose (Xyl), d-quinovose (Qui), 3-*O*-methyl-d-xylose (MeXyl) [59].

Triterpene glycosides also called triterpene saponins are secondary metabolites typically produced by all sea cucumbers. Nowadays, the number of triterpene glycosides isolated from sea cucumbers exceeds 300. From a chemical point of view, these glycosides are amphiphilic compounds that have aglycone (lipophilic) and glycone (hydrophilic) moietes. The majority of the triterpene glycosides contain the so-called holostane type aglycone comprising of lanostane-3β-ol with a γ(18,20)-lactone in the E-ring of the pentacyclic triterpene [3β,20S-dihydroxy-5α-lanostano-γ(18,20)-lactone] (Figure 4). Some glycosides contain a non-holostane type aglycone which do not have γ(18,20)-lactone in the tetracyclic triterpene. Generally, the triterpene glycosides consist of two to six monosaccharide units such as d-xylose, d-quinovose, d-glucose, 3-*O*-methyl-d-glucose, 3-*O*-methyl-d-xylose (Figure 5) and rarely 3-*O*-methyl-d-quinovose, 3-*O*-methyl-d-glucuronic acid and 6-*O*-acetyl-d-glucose covalently linked to C-3 of the aglycone. The sugar units of the glycone part are generally arranged in linear or branched chains (Figure 6). In addition, these chains may carry from one to three sulfate groups. About sixty percent of the sea cucumber triterpene glycosides have sulfate groups linked to the monosaccharide units of the carbohydrate chain. The chemical structures of 341 glycosides isolated from more than 50 species of holothurians are provided in the review article of M.A.M. Mondol et al. [59].

It is very interesting that triterpene glycosides from sea cucumbers exert a wide spectrum of cytotoxic effects affecting various cells under in vitro conditions, in particular, some types of human tumor cells, such as U-87-MG, HCT-8, P-leukemia 388, КБ, Schabel, Mel-28, А-549, MICF-1, НТ-29, IA9, Caki-1, СК-MEL, ПК-3, lymphoid leukemia L 1210, MCF-7, MKN-28, НСТ-116, U87MG, Hep-G2, HeLa cells, ТНР-1, КБ-VIN, НСТ-8, C33A, and others [60] (Table 5).

Argusides A, B, C, D and E were isolated from the sea cucumber *Bohadschia argus*. They were shown to exert high cytotoxic activity against various human tumor cell lines. For example, arguside A dramatically affects human colorectal carcinoma (HCT-116) cells (IC_50_ = 0.14 μM) [61]. Argusides B and C also demonstrate potent efficacy against adenocarcinomic human alveolar basal epithelial cells (A549), HCT-116, Hep-G2, and human breast adenocarcinoma (MCF-7) cell lines with IC_50_ values of 0.48 and 0.43 μg/mL, respectively, on A549 with 0.46 and 0.38 μg/mL, respectively, on HCT-116 [62]. Argusides D and E were shown to be very effective regarding all aforementioned human tumor cell lines [63]. Bivittoside D whoch found in several species of sea cucumber belonging to the family *Holothuriidae* was found to have almost the same activity [64]. A chemical analogue of bivittoside D named pervicoside C has the same aglycone part but different sugar chain and was isolated from *Holothuria fuscocinerea*. Surprisingly, this glycoside showed very weak activity against HCT-116 and A549 cancer cells (IC_50_ 18.7 and 28.6 μg/mL, respectively [64]. Another species of sea cucumbers, *Cercodemas anceps*, family Cucumariidae, contains triterpene glycoside colochiroside A, which was registered under in vitro conditions to affect several types of the tumor cell lines including P-388, HL60, A-549, SpC-A4, MKN28, and SGC-7901. In animal studies with mice colochiroside A significantly inhibited growth of H22 liver cancer cells and S180 sarcoma cells [65].

Hillaside C, a lanostane-type triterpene glycoside from *Holothuria hilla* possesses cytotoxic properties with IC_50_ values varying from 0.15 to 3.20 μg/mL against several human tumor cell lines (A-549, MCF-7, human lung carcinoma cells-IA9, human clear cell carcinoma cells CAKI-1, human prostate cancer cells PC-3, KB, KB-VIN, and human colorectal adenocarcinoma cells HCT-8) [77]. Another lanostane-type impatienside was isolated from *Holothuria impatiens* and showed higher cytotoxic activity in vitro regarding human tumor cell lines A549 (IC_50_ values of 0.35 and 0.52 μg/mL), HCT-116 (IC_50_ 0.45 and 0.37 μg/mL), DU-145 (IC_50_ 1.14 and 0.937 μg/mL), and KB (IC_50_ 1.6 and 1.42 μg/mL, respectively) than common antitumor drug etoposide [64]. Much higher cytotoxic activity regarding human tumor cell lines, A549 (IC_50_ 0.886 and 1.07 μM), HL-60 (IC_50_ 0.245 and 0.427 μM), BEL-7402 (IC_50_ 0.97 and 1.114 μM), and human acute lymphoblastic leukemia cell line (Molt-4) (IC_50_ 0.34 and 0.521 μM) was found in a couple of specific triterpene glycosides isolated from *Holothuria grisea* and named 17-dehydroxyholothurinoside A and griseaside A [76]. Triterpene glycosides isolated from *Holothuria fuscocinerea* namely fuscocinerosides A, B and C, pervicoside C and holothurin A were shown to possess more pronounced cytotoxic activity against human leukemia HL-60 and human hepatoma BEL-7402 cells than fuscocineroside C (IC_50_ = 0.88 and IC_50_ = 0.58 μg/mL, respectively). Structurally, all these compounds are very similar as they have 3-*O*-methyl-β-d-glucopyranosyl-(1→3)-β-d-glucopyranosyl-(1→4)-β-d-quinovopyranosyl-(1→2)-4-*O*-sodiumsulfato-β-d-xylopyranosyl linked to C-3 of the holostane triterpene aglycones with slight differences in their side chain patterns and 17-substituents [75]. Holothurins A3 and A4 were isolated from the sea cucumber *Holothuria scabra* and under in vitro conditions they showed strong cytotoxic effects towards several human tumor cell lines such as epidermoid carcinoma (KB) and hepatocellular carcinoma (Hep-G2), with IC_50_ values of 0.87 and 0.32 μg/mL (for A3) and of 1.12 and 0.57 μg/mL (for A4), respectively [78].

Almost all triterpene glycosides found in sea cucumbers have a carbohydrate chain with quinovose as the second consecutive unit. Glucose is rarely observed at this position. For example, sulfated glycoside philinopside E from the body wall of *Colochirus quadrangularis* has the aforementioned quinovose pattern, but non-sulfated philinopside F obtained from the same sea cucumber is completely devoid of quinovose. Comparison of cytotoxic activity of these two compounds showed that non-sulfated philinopside E is effective against ten tumor cell lines (gastric carcinoma cells MKN28, mouse lymphocytic leukemia cells P388, BEL7402, HL60, lung adenocarcinoma cells SPC-A4, A549, gastric carcinoma cells SGC7901, human ovarian carcinoma HO8901, human fetal lung fibroblasts W138, human epithelial carcinoma cells A431) with an IC_50_ = 0.75–3.50 μg/mL. Sulfated glycoside philinopside E was not effective at all [69]. At the same time, sulfated triterpene glycosides philinopsides A and B obtained from sea cucumber *C. quadrangularis* were shown to cytotoxically affect such tumor cell lines as P-388, A549, MCF-7, MKN28, HCT-116, and U87MG with IC_50_ = 0.60–3.95 μM [70]. Philinopsides A and E were also found to induce apoptosis of mouse Sarcoma-180 tumor cells as well as tumor-associated endothelial cells resulting in the reduction of the total tumor node volume. It was noted that these triterpene glycosides inhibited virtually all receptor tyrosine kinases related to angiogenesis [71,72]. Another three triterpene glycosides violaceusides I, II and III isolated from the sea cucumber *Pseudocolochirus violaceus* showed high cytotoxic activity against stomach adenocarcinoma MKN45 and HCT-116 with IC_50_ values in the range of 0.068–0.352 μM. These holothurian species also contain sulfated triterpene glycoside intercedenside B that exhibit pronounced cytotoxicity against MKN45 and HCT-116 with an IC_50_ = 0.052–0.442 μM. Moreover, violaceuside I and intercedenside B possess markedly higher cytotoxic activity against MKN45 and HCT-116 in comparison to the hexachloroplatinate used as a positive control [87].

Pentactasides I, II and III belonging to the group of holostane-type triterpene glycosides were obtained from the sea cucumber *C. quadrangularis*. They are characterized by slightly different structures because pentactasides I and II have the same trisaccharide pattern (3-*O*-[β-d-xylopyranosyl-(1→4)-β-d-chinovopyranosyl-(1→2)-4-*O*-sulfo-β-d-xylopyranosyl]) that is a rare chemical feature among naturally occurring sea cucumber glycosides, whereas pentactaside III is a typical sulfated diglycoside. All these triterpene glycosides cytotoxically affected several malignant cell lines such as P-388, A-549, MCF-7, MKN28, HCT-116, and U87MG with IC_50_ varying from 0.60 to 3.95 μM [82].

The sea cucumber *Psolus patagonicus* contains patagonicoside A reducing the growth rate of three tumor cell lines, namely Hep3B, MDA-MB231, and A549 [81].

The sea cucumber *Pearsonothuria graeffei* was found to contain non-sulfated triterpene glycoside ds-echinoside A, which had complex effects against malignant tumors. It can reduce viability of the human liver carcinoma cells Hep-G2, inhibit adhesion, migration, and invasion of Hep-G2 cells in a concentration-dependent manner, inhibit matrix metalloproteinase-9 (MMP-9) expression, increase expression of metalloproteinase-1 tissue inhibitors and also reduce the expressions of vascular endothelial growth factor (VEGF) and NF-κB [69]. Furthermore, later studies showed that ds-echinoside A increases expression of cell-cycle-related genes such as p16, p21, and c-myc, and inhibits expression of cyclins D1 and B-cell lymphoma 2 (Bcl-2) associated with accelerated release of mitochondrial cytochrome, activated caspase-3 and cleavage of adenosine diphosphate ribose polymerase. In animal studies ds-echinoside A contributed to significant reduction of the H22 hepatocarcinoma tumor weight [68].

Holothurin A and 24-dehydroechinoside A are sulfated triterpene glycosides from the body wall of sea cucumber *Pearsonothuria graeffei*. They markedly decrease expression of MMP-9, increase expression of TIMP tissue inhibitors and almost completely abolish VEGF expression under in vitro and in vivo conditions resulting in significant inhibition of the metastasis process [70].

Holothuria *Mensamaria intercedens* contains a group of glycosides generally termed intercedensides. In experiments with several human tumor cell lines (A549, MCF-7, IA9, CAKI-1, human glioblastoma cells U-87-MG, PC-3, KB, KB-VIN, human skin melanoma cells SK-MEL-2 and HCT-8) intercedensides A, B, and C showed cytotoxic effects. In mice experiments with Lewis lung carcinoma and S180 sarcoma intercedenside A dramatically suppressed growth of tumor nodes [70].

Stichoposide C isolated from the body wall of the sea cucumber *Thelenota anax* was shown to dose-dependently induce apoptosis in human leukemia and colorectal cancer cells following activation of Fas and caspase-8, cleavage of Bid, mitochondrial damage, and caspase-3 activation. In addition, under in vivo conditions, growth of the HL-60 xenograft and CT-26 subcutaneous tumors was significantly decreased [86].

Eight triterpene diglycosides, including four new compounds named stichorrenosides A, B, C and D and several already known compounds, stichoposides A and B, 3β-*O*-[β-d-xylopyranosyl-(1→2)-β-d-xylopyranosyl]-23S-acetoxyholost-7-ene, and 3β-*O*-[β-d-xylopyranosyl-(1→2)-β-d-xylopyranosyl]-23S-hydroxyholost-7-en were isolated from the sea cucumber *Stichopus horrens*. They influence the human cancer cell lines such as epidermoid carcinoma (KB), hepatoma cancer (Hep-G2), breast cancer (MCF7), prostate cancer (LNCaP), and melanoma (SK-Mel2) in vitro. The results showed that the highest cytotoxicity against all these tumors is demonstrated by stichorrenoside D, stichoposide A, and 3β-*O*-[β-d-xylopyranosyl-(1→2)-β-d-xylopyranosyl]-23*S*-acetoxyholost-7-ene with IC_50_ values ranging from 1.92 ± 0.61 to 3.13 ± 0.40 μM, from 2.12 ± 0.30 to 3.02 ± 0.33 μM and from 2.04 ± 0.73 to 3.11 ± 0.32 μM, respectively. Their activity was close to that of antitumor drug ellipticine (IC_50_ values ranging from 1.34 ± 0.16 to 1.95 ± 0.20 μM) used as a positive control. Stichorrenoside C and stichoposide B also revealed marked efficacy against all five tumor cell lines. Other mentioned triterpene glycosides demonstrated moderate cytotoxicity. Evaluation of the relationships between structure and cytotoxic activity of these active compounds suggested that the crucial factor is the presence of quinovose and the second xylose residues in the disaccharide chains as well as acetoxyl groups in side chains [9].

Frondoside A obtained from the Atlantic sea cucumber *Cucumaria frondosa* inhibited cell proliferation of the AsPC-1 human pancreatic cancer cell line in a concentration- and time-dependent manner. Immunohistochemical studies revealed that such activity was caused by increased sub-G0/G1 apoptotic cell population, decreased Bcl-2 and Mcl-1 expression, increased Bax expression and expression of the cyclin-dependent kinase inhibitor p21 as well as activation of caspases 3, 7, and 9. In animal studies with xenograft mouse models frondoside A was used in a very low dose 10 μg/kg/day resulting in inhibited growth of AsPC-1 [72]. Results of another study showed that frondoside A if administered intraperitoneally showed marked antimetastatic influence on the syngenic murine model of metastatic breast cancer and reduced the number of spontaneous tumor metastasis in mouse lungs with mammary gland-implanted tumors [88]. Frondoside A at IC_50_ concentrations of 0.7–2.5 μM also reduced viability of MCF-7, NCI-H460-Luc2, A549, MDA-MB-435, Hep-G2, and LNM35 within 24 h as well as induced the caspase 3/7-dependent apoptosis pathway in a concentration-dependent manner. Daily intraperitoneal injections of 0.01 and 1.0 mg/kg of frondoside A for 25 days significantly inhibited the growth, angiogenesis, and lymph node metastasis of LNM35 xenograft cells in athymic mice. It was found that frondoside A can inhibit the PAK1-dependent growth of A549 lung cancer cells with IC_50_ from 2.5 μM in 24 h to 0.6 μM in 72 h. Besides, under in vitro conditions, it directly inhibits the oncogenic/ageing kinase PAK1 at an IC_50_ of about 1 μM. At the same time, it slightly affects two other oncogenic kinases, LIMK and AKT at an IC_50_ of about 60 μM [73,74]. Frondoside glycosides were tested in a comparative study. The results showed that frondoside A inhibits growth of pancreatic cancer cells at EC_50_ ~ 1 μM whereas frondoside B is less effective (EC_50_ ~ 2.5 μM) and frondoside C while its aglycone does not exert any cytotoxic effect. In an animal study, frondoside A was administered intraperitoneally in a dose of 100 μg/kg/day leading to significantly inhibited growth of tumor xenografts in nude mice CD2F1. The same dose administered via gastric gavage did not exert any effect. In this study the main pharmacokinetic characteristics of oral frondoside A administration were obtained as follows: maximum plasma concentration (Cp_max_)—129 nM, bioavailability—20%, total body clearance (Cl_tb_)—6.35 mL/min/m^2^, and half-life period −510 min. Pharmacokinetics of intraperitoneal administration was following: Cp_max_—18.3 nM, bioavailability—100%, Cl_tb_—127 mL/min/m^2^ and half-life period—840 min [89].

Activity of triterpene glycosides cucumariosides A2-2 and A4-2 isolated from sea cucumber *C.f. japonica* were compared to frondoside A. All these compounds induced apoptosis in human leukemia cells HL-60, THP-1, NB-4 and K562 in vitro via caspase-dependent mechanisms. Frondoside A was shown to exert faster and more potent apoptosis than cucumariosides [80].

Wang J. et al. [67] isolated nine triterpene glycosides from *Holothuria scabra* namely echinoside A, 24-dehydroechinoside A, HS-1, holothurins B, B4, A and A1, scabrasides D and B, and four glycosides from the sea cucumber *Cucumaria frondosa* such as frondosides A1, A and A6 and 24-dehydrofrondoside A6. Their cytotoxic activity against human tumor cells including human hepatoma (Hep-G2), human cervical cancer (HeLa), and human leukemia (K562) cells were investigated. The most potent apoptosis-inducing activity was noted in experiments with echinoside A and Hep-G2 cells. Detailed studies showed that this compound markedly decreased mitochondrial transmembrane potential and Bcl-2/Bax mRNA express ratio. At the same time, it p-regulated the mRNA expression levels of caspase-3, caspase-8 and caspase-9 in Hep-G2 cells [67]. The sea cucumber *Holothuria scabra* was found to contain a variety of triterpene glycosides, three of them were identified as scabraside D, fuscocineroside C, and 24-dehydroechinoside A and tested in vitro for their anti-proliferative activity regarding such tumor cell lines as mouse leukemic cells (P-388), lung human cancer cell (A-549), human colorectal cancer cell (HCT-116), gastric cancer cell (MKN28), and human breast cancer cell (MCF-7). The results confirmed that all three glycosides exert significant cytotoxicity [90]. The following studies gave more promising data confirming significant activity of scabraside D, which reduced viability and migration of the human cholangiocarcinoma cells in a dose-dependent manner, with an IC_50_ of 12.8 ± 0.05 μg/mL at 24 h. The study also registered the signs of apoptotic cells with decreased Bcl-2 and high levels of Bax and caspase-3 gene expression. Comparison of effects exerted by scabraside D and antitumor drug 5-fluorouracil on cell viability showed no significant differences when they were administered at the same concentration. In animal studies, scabraside D was injected in a 1 mg/kg/day dose for three weeks to mice and contributed significantly reduced growth rate of human cholangiocarcinoma xenografts with no adverse effects on the BALB/cMlac-nu mice [91].

Sulfated saponins holothurin A, moebioside A, holothurin B and 24-dehydroechinoside B isolated from the whole body of the sea cucumber *Holothuria moebii* significantly reduced proliferation rate of human colorectal cancer cells HCT-8, HCT-15, HCT-116, and SW620 with IC_50_ values varying from 1.04 to 4.08 μM and provoked late apoptosis of HCT-15 cells at the beginning of treatment at 3 μM concentration. Furthermore, these saponins showed cytotoxic activity against colorectal tumor cells that is almost similar to that of doxorubicin. Intraperitoneal administration of a complex of these glycosides in a 120 mg/kg/day dose suppressed the growth of colorectal CT-26 tumor-bearing Balb/c xenografts in mice [92]. In another experiment, they inhibited proliferation of four types of glioma cells with an IC_50_ ranging from 0.99 to 8.64 μM in a dose-dependent manner. Moreover, moebioside A isolated from *H. moebii* lowered the expression of metabolic enzymes of glycolysis and glutaminolysis in human glioblastoma U87-MG cells and induced apoptosis [71,80].

Current experimental structural and functional interactions data of triterpene glycosides suggest that the length and type of sugar moieties play an important role in cytotoxic activity against human tumor cells.

The data presented in this section show that many triterpene glycosides found in sea cucumbers possess high cytotoxic activity against cancer cells. Antitumor activity of this group of natural compounds is very close and, sometimes, significantly higher, to that of approved anticancer pharmaceuticals such as doxorubicin and some others. The wide variety of cancer cell types that are sensitive to the influence of triterpene glycosides looks very promising. Some selected glycosides like echinoside A, ds-echinoside A, frondoside A and cucumarioside A2-2 were shown to have comprehensive mechanisms of their cytotoxic effects against cancer cells associated with inhibition of cell cycle progression through activation of the apoptosis pathway leading to cell death. Glycosides were shown to activate intrinsic apoptotic pathways by suppression of the tumor gene, p53 suppressor, resulting in the up-regulation of the caspases 3, 7, 8 and 9 and activation of the cell death process [66,68].

### 3.2. Cerebrosides

**Figure 7:**
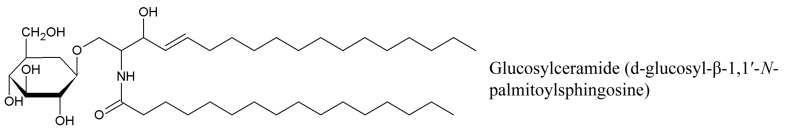
Representative structure of glucosylceramide (d-glucosyl-β-1,1′-N-palmitoylsphingosine).

**Figure 8:**
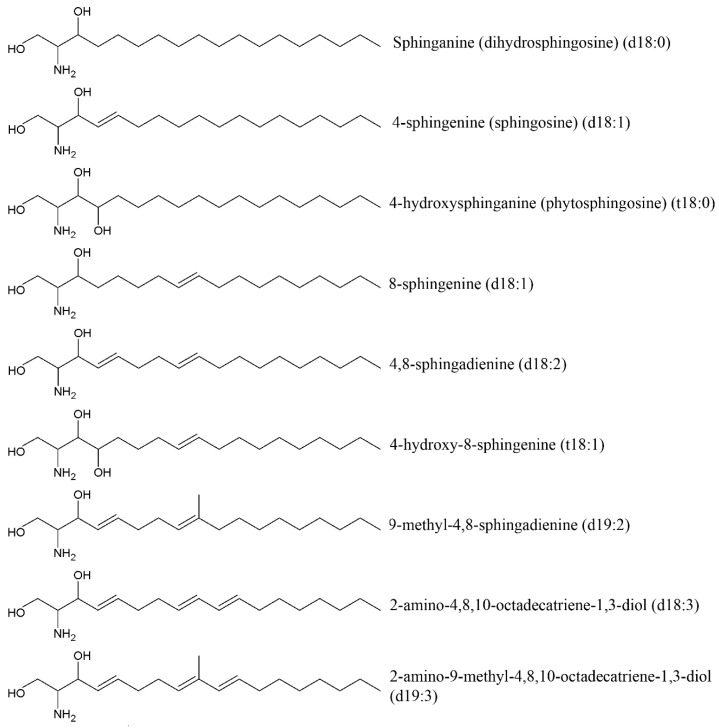
Structures of naturally occurring sphingoid bases.

Cerebrosides, neutral glycosphingolipids, are composed of two different structural units: hexose (glucose for glucocerebrosides or galactose for galactocerebrosides) and ceramide constituted of a sphingoid base, also called a long-chain base (LCB), and an amide-linked fatty acid (FA). Ceramide formation occurs through a β-glycoside bond between the hydroxide radical at C-1 of ceramide and hexose [93] (Figure 7). The most common sphingoid base of mammalian sphingolipids is sphingosine (4-sphingenine, d18:1) usually attached to a branched 2-OH or non-hydroxyl alkyl within 16–24 carbon atoms. Sphinganine (dihydrosphingosine, d18:0), phytosphingosine (4-hydroxysphinganine, t18:0) and some others are often found in small amounts. Sphingoid bases from plants include d18:1, d18:2, and 4-hydroxy-8-sphingenine (t18:1) (Figure 8).

Sphingoid bases in marine invertebrates have been reported to be significantly different from those found in plants and animals. Sphingolipids of marine invertebrates have a unique triene type of sphingoid bases with a conjugated diene such as 2-amino-4,8,10-octatriene-1,3-diol (d18:3) and 2-amino-9-methyl-4,8,10-octatriene-1,3-diol (d19:3). The first studies devoted to investigating holothurian sphingolipids indicated that three types of major LCBs of the sea cucumber cerebrosides (d17:1, d18:2, and t17:1) isolated from *Apostichopus japonicus*, *Cucumaria frondosa* and *Acaudina molpadioides* [94,95]. Sugawara et al. [96] found d17:1, d18:1, d18:2, d18:3, d19:1, d19:2 and d19:3 in the sea cucumber *S. horrens*. Thirteen LCBs from the sea cucumber *Pearsonothuria graeffei* were analyzed and shown to have a d17:1 (37.88%), d18:1 (21.52%), d18:2 (11.44%), d18:3 (6.08%), t17:0 (6.37%). d17:1 is a predominant LCB among the sea cucumber cerebrosides [97]. Generally, it is not found in mammals, plants, or fungi.

The fatty acid moieties of the sea cucumber cerebrosides are usually saturated (mostly C18:0, C20:0, C22:0, C24:0), monounsaturated (mostly C20:1, C22:1, C24:1), and α-hydroxyl fatty acids (mostly C22:0h, C23:0h, C23:1h, and C24:1h) with about twenty fatty acids total. In most analyzed holothurians the glycosyl group is a glucose unit whereas galactose is found only in the sea cucumber *Bohadschia argus* [98]. It is obvious that modification in chain length and degree of saturation and/or hydroxylation of LCBs and FAs leads to extensive variation of the cerebroside structure. Eighty-nine cerebrosides were identified in the sea cucumber *P. graeffei* using liquid-chromatography-quadrupole-time-of-flight-mass-spectrometry with large amounts of d17:1–C22:0h, t17:0–C24:1h, d17:1–C24:1h, d17:1–C23:0h, d17:1–C22:0 and d17:1–C23:0 rarely found in mammals [97].

Investigation of the antitumor potential of cerebrosides showed that such long-chain bases as d17:1, 4, 8-sphingadienine (d18:2), d18:3 and d19:3 inhibit proliferation of human colonic cancer cells DLD-1, WiDr and Caco-2 in a dose-dependent manner due to induction of apoptosis [96] (Table 6).

Similar molecular compounds belonging to glucocerebrosides were extracted from *Cucumaria frondosa*. Their structural characteristics were as follows: the fatty acid part was usually saturated (C22:0 and C18:0), monounsaturated (C24:1 and C20:1) and α-hydroxyl fatty acids (C24:1h, C23:0h, C23:1h and C22:0h), sphingoid base consisted of dihydroxy (d17:1, d18:2 and d18:1) and trihydroxy (t17:0 and t16:0) chains and glucose was attached as monosaccharide moiety. Composition analysis of the long-chain bases showed the ratio of d18:2 and d17:1 was close to 2:1. Long-chain bases and glucocerebrosides were found to have in vitro cytotoxic activity against Caco-2 colon cancer cells [99]. Cerebrosides isolated from sea cucumber *Acaudina molpadioides* were composed of glucose, amide-linked fatty acid consisting of C18:0 h, C22:1 h, C23:1 h or C24:1 h and the sphingoid base was d17:1. This type of cerebrosides in in vitro experiments inhibited proliferation of the sarcoma S180 cells by inducing apoptosis. Administration of these cerebrosides into mice with implanted S180 cells in a 50 mg/kg body weight dose led to reduction of tumor weight. It was found that cerebrosides down-regulated expression of Bcl-2 and Bcl-xL and up-regulated expression of Bax, cytochrome C, caspase-9 and caspase-3 mRNA level in the S180 ascites tumor cells [100]. Isolated sphingoid bases composed of 4,8,10-sphingatrienine (d18:3) and 9-methyl-4,8,10-sphingatrienine (d19:3) induced apoptosis in human hepatoma Hep-G2 cells via up-regulation of apoptosis-inducing death receptor-5 (DR5), growth arrest DNA damage protein growth arrest and DNA damage gene 45, apoptosis inducer protein Bax and the peroxisome proliferator-activated receptor-γ (PPARγ) and down-regulation of p-AKT resulting in reduced tumor cell viability [101].

### 3.3. Fucosylated Chondroitin Sulfates

Native FuCS was isolated from sea cucumber *Cucumaria frondosa* and used to prepare low-molecular-weight FuCS. The latter was given to male C57BL/6 mice with implanted Lewis lung carcinoma and led to inhibition of tumor growth and intensity of metastasis in a dose-dependent manner. The low-molecular-weight FuCS increased p53/p21 expression and apoptosis due to enhanced activity of caspase-3 resulting in cell cycle arrest in Lewis lung carcinoma cells. Its mechanism of antiproliferative influence is related to suppressed expression of vascular endothelial growth factor, increased expression of tissue inhibitor of metalloproteinase-1 and down-regulation of MMPs as well as significantly reduced activity of the extracellular signal-regulated protein kinase 1/2/p38 mitogen-activated protein kinase/NF-κB pathway which plays the leading role in expression of MMPs [31]. These results suggest that low-molecular-weight FuCS may be considered a promising anti-tumor drug candidate.

FuCS was shown, in animal studies with mice, to reduce pulmonary metastasis of B16F10 melanoma cells because it inhibits P-selectin-mediated adhesion of the tumor cells to platelets and tumor cell migration demonstrated by specific in vitro experiments. This key element of glycosaminoglycans antimetastatic influence results in dramatic reduction of P-selectin-mediated adhesion of tumor cells and in down-regulation of protein levels of such integrins as focal adhesion kinase and MMP-2/9 in B16F10 cells [10]. Specific glycosaminoglycan isolated from sea cucumber *Holothuria leucospilota* has the capacity to inhibit thrombin-induced platelet activation and aggregation of platelets, reduce adhesion of the breast cancer cells to platelets and prevent adherence of the platelet-cancer cell complex to fibrinogen, weaken formation of the complexes between platelets and cancer cells and suppress mRNA and protein levels of β1 and β3 integrins, MMP-2 and MMP-9. It can also increase expression of MMP inhibitor and tissue inhibitor of metalloproteinase-1 in MDA-MB-231 cells. Thus, the mechanism of antitumor activity of holothurian glycosaminoglycans is directly dependent on its antiplatelet properties and capacity to inhibit cellular adhesion between tumor and normal cells [102].

## 4. Antiprotozoal, Antibacterial, Antiviral Agents

Biologically active compounds isolated from sea cucumbers have more or less pronounced pharmacological activity against pathogens inducing invasive and infectious diseases (Table 7).

### 4.1. Antimalarial Agents

One of the most hazardous problems in the treatment of infectious diseases is drug resistance, which develops in almost all human pathogens reducing efficacy of treatment and posing serious threats to public health. That is why the search for new antimicrobial agents from various natural sources is always important and marine species are considered the most promising ones. Administration of glycosaminoglycans, in particular, heparin, have been proposed to prevent malaria as they can inhibit cytoadhesion of the malaria plasmodium to the capillary and postcapillary endothelium cells as well as to placental trophoblasts, block invasion of parasites and disrupt rosettes, the morphological phenotype of plasmodium progression forming the binding of infected erythrocytes to noninfected ones. Unfortunately, heparin possesses various adverse effects including a high risk of contamination because most glycosaminoglycans are isolated from mammal species. Therefore, the use of the heparin-based therapy is quite difficult. One possible solution may be using compounds with similar pharmacological properties from other sources, such as sea cucumbers.

Investigation of the effects exerted by native FuCS isolated from the body wall of the sea cucumbers *H. grisea* on the life circle of *Plasmodium falciparum* showed that its glycosaminoglycans significantly inhibited cytoadhesion of cultured parasite-infected erythrocytes to human lung endothelial cells and placenta cryosections. In addition, FuCS interferes with merozoite invasion resulting in disruption of rosettes and blocked plasmodium development. The inhibiting activity of FuCS can be abolished by removing sulfated fucose branches. It should be pointed out that FuCS applied in concentrations 1 μg/mL and lower is more effective than heparin. The chondroitin sulfate mechanism of action very similar to that of heparin blocks parasite invasion, adhesion, and rosetting by binding to regions of Plasmodium falciparum erythrocyte membrane protein 1 and merozoite surface protein-1 (MSP-1).

FuCS very likely attaches to conserved regions of host receptors for constitutively expressed glycoproteins in erythrocytes preventing adhesion of proteins to these receptors and thus blocking their invasion through the membrane [103].

Marques et al. [6] have explored the antiplasmodial capacity of sulfated polysaccharides from the sea cucumbers *H. grisea* and *Isostichopus badionotus*. The main structural pattern of the FuCS isolated from *I. badionotus* is a simple branched α-fucose disulfated at positions 2 and 4 (about 90%) or exclusively 4-sulfated (about 10%). FuCS obtained from *H. grisea* was shown to have a more complicated structure, generally composed of disaccharide α-fucose units non-sulfated and 3-sulfated at the reducing and nonreducing ends, respectively. This FuCS also contains a few branches formed by single α-fucose units disulfated at positions 2 and 4 (about 27%) or 2 and 3 (about 20%). The linear sulfated fucans from these sea cucumbers consist of numerous tetrasaccharide sequences, defined by patterns of sulfation at positions 2 and 4, which differ exclusively in sulfation of the second residue of the tetrasaccharide. Fucans from *I. badionotus* are generally 2-sulfated whereas the ones from *H. grisea* are non-sulfated. Average molecular weight of the FuCS is about 30–40 kDa whereas fucans are characterized by molecular weight close to 200 kDa. Under in vitro conditions FuCS isolated from *H. grisea* showed pronounced antimalarial effects via inhibition of plasmodium development with IC_50_ 2.3 µg/mL. IC_50_ of FuCS from *I. badionotus* was 4.2 µg/mL. Fucans from these two sea cucumbers were significantly less effective with IC_50_ for *L. grisea* of 20.3 µg/mL and for *I. badionotus*—of 9.5 µg/mL. Such activity of these natural compounds is very close to heparin activity varying from 4.1 to 18.0 µg/mL depending on the batch used [6].

These results suggest that FuCS as sulfated fucans isolated from marine invertebrate sea cucumbers may be a supplementary therapy for some severe malarial complications. The main advantages of these substances are the possibility of using them with no chemical modifications and their source is not a mammalian species. These advantages dramatically reduce the risk of pathogen contamination. The body wall of various sea cucumbers contains a large amount of these compounds making it possible to isolate them in relatively high yields. They exert anticoagulant and antiplatelet activities much weaker than those of heparin. The concentration required for antimalarial effects is too low to induce anticoagulant cascades. FuCS and sulfated fucans were shown in animal studies to be toxicologically safe with no signs of accumulation even when given daily in large doses. The oral route of administration is another big advantage for these compounds [6,103].

### 4.2. Anti-Leishmania Agents

A methanol extract and the n-butanol fraction obtained from sea cucumber *Actinopyga lecanora* demonstrated antiprotozoal activity against leishmaniosis pathogen *Leishmania donovani*. The *n*-butanol fraction obtained from the sea cucumber contained two types of glycosides, holothurin B exerting powerful leishmanicidal activity under in vitro conditions and moderate in vivo, and holothurin A showing only slight influence on the pathogen [104]. Methanol extract of the body wall and coelomic fluid of *Holothuria leucospilota* was shown to exert strong antileishmanial activity in experiments with *Leishmania infantum* tested using both promastigote and axenic amastigote forms. Coelomic fluid was shown to be the most active agent against promastigotes and amastigotes with IC_50_ of 62.33 and 22.4 μg/mL at 48 h and 73 and 46 μg/mL at 72 h after treatment, respectively [110]. Despite these sparse results, sea cucumbers may be used to discover new pharmaceutical prototypes with antileishmanial activity.

### 4.3. Antibacterial and Antifungal Agents

According to numerous studies, sea cucumbers contain biologically active compounds with either very low or even no antibacterial properties regarding many common pathogens. For example, a number of experiments focused on exploring ethyl acetate, methanol, or water-methanol extract obtained from the body wall, coelomic fluid or cuvierian organs of sea cucumbers. It was found out that these extracts do not kill or prevent proliferation of *Pseudomonas aeruginosa*, *Escherichia coli*, *Staphylococcus aureus* and some other pathogens [111]. Some studies showed moderate antibacterial activity of holothurian compounds when they were applied in very high concentration. Weak bacteriostatic activity against human pathogens was noted in experiments with hydroalcoholic, n-hexane, chloroform, and methanol extracts obtained from different tissues and organs of sea cucumber *Holothuria leucospilota* used in concentrations of 1000 and 2000 μg/mL. Even such high concentrations did not result in bacteriostatic effects [112]. Similarly, ethanol extracts of sea cucumbers *Actinopyga echinites*, *Actinopyga miliaris*, *H. atra* showed slight antimicrobial activity against some bacterial strains such as *E. coli*, *Aeromonas hydrophila*, *Enterococcus* sp., *Pseudomonas aeruginosa, Klebsiella pneumoniae*, *S. aureus, Salmonella typhi* and *Vibrio harveyi* [111]. Extracts of *H. scabra* were tested on many bacterial strains with no signs of bacterial inhibition regardless extract concentration [113,114]. The absence of antibacterial activity of the *H. scabra* extracts against *S. aureus* and E. coli were confirmed by studies testing compounds obtained from *H. leucospilota* in the Persian Gulf [115].

In some studies protein hydrolysates from holothurians by enzyme hydrolysis of the fresh sea cucumbers were explored for antibacterial activity against pathogenic Gram positive and Gram negative strains. The result showed quite low efficacy for pure holothurian peptides used as antibacterial agents in comparison with common synthetic antibacterial drugs [116]. At the same time, protein hydrolysates from sea cucumbers are considered a promising non-toxic and safe alternative natural source to chemical food preservatives.

Despite their low antibacterial activity, holothurian extracts were shown to possess activity regarding fungal pathogens. A crude methanol extract of *Actinopyga lecanora* demonstrated marked antifungal activity against *Candida albicans* (minimum inhibitory concentration, MIC, 62.5 μg/mL), *C. neoformans* (MIC, 125 μg/mL), *Sporothrix schenckii* (MIC, 62.5 μg/mL), *Trychophyton mentagrophytes* (MIC, 125 μg/mL) and *Aspergillus fumigatus* (MIC, 31.2 μg/mL). Methanol extracts of these sea cucumbers were found to contain three active triterpene glycosides, namely, holothurin B, holothurin A and another new compound. Comparative studies have shown that holothurin B exerts more pronounced antifungal effects inhibiting *Trychophyton mentagrophytes* proliferation more than the commonly used specific antifungal drug fluconazole. Furthermore, holothurin B is characterized by MIC as very close to fluconazole in inhibiting the growth of *Sporothrix schenckii* and *Aspergillus fumigatus*. The other two triterpene glycosides were not found to possess any antifungal properties [112]. Holothurin B may be a lead prototype for development of new safe antifungal agents.

This deduction was confirmed by the significant activity of holostan-type triterpene glycosides, marmoratoside A, 17α-hydroxyimpatienside A, impatienside A and bivittoside D isolated from sea cucumber *Bohadschia marmorata* against six strains of fungi with MIC_80_ varying from 0.70 to 2.81 µM. For example, bivittoside D when tested alone showed antifungal activity that was comparable to that of the standard antifungal drug ketoconazole against *C. albicans* (MIC 0.78 μg/mL), *T. mentagrophytes* (MIC 0.78 μg/mL) and *A. fumigates* (MIC 1.56 μg/mL) [106,107].

The triterpene glycosides holotoxins B, A and A1 isolated from A. japonicus exerted fungicidal effect against various Candida species including several clinical isolates, although its antifungal activity was lower than that of amphotericin B and miconazole by an average of 17.5–23 times [108].

### 4.4. Antiviral Compounds

Some recent studies with the compounds isolated from sea cucumbers have shown their antiviral potential. The water extract of *Holothuria* sp. (from the Persian Gulf) exerted significant activity against herpes simplex virus type 1 (HSV-1) in cell culture at half maximal cytotoxic concentration (CC_50_) 32.57 mg/mL and IC_50_ inhibition of virus adhesion to cells and intracellular replication of 120.2 and 189.9 μg/mL, respectively [117].

FuCS obtained from sea cucumber *Thelenota ananas* effectively inhibited entry and replication of the laboratory strain HIV-1_IIIB_ (4.26 and 0.73 μg/mL, respectively), prevented infection of clinical isolates HIV-1_KMO18_ and HIV-1_TC-2_ (23.75 and 31.86 μg/mL, respectively), suppressed HIV-1 drug-resistant virus and inhibited HIV-2_ROD_ and HIV-2_CBL-20_ replication (100 μg/mL). It should be emphasized, that this FuCS showed high antiviral activity against T-20-resistant strains with EC_50_ values between 0.76 μg/mL and 1.13 μg/mL. It was clearly shown that FuCS can bind the recombinant HIV-1 gp120 protein, but inhibition of recombinant HIV-1 reverse transcriptase was not observed. These results suggest FuCS may have potential to be further developed as a novel HIV-1 entry inhibitor for treatment of HIV/AIDS patients [109].

## 5. Antioxidants

Mechanisms of many pathogenic disorders are often linked to the damaging influence on cell membranes, proteins, lipids, DNA and other biomolecules with unpaired electrons referred to as free radicals such as reactive oxygen species (ROS) and reactive nitrogen species (RNS). All individual cells as well as whole organisms possess their own protective systems that scavenge free radicals reducing their damaging effects and repair cellular damage using compounds known as antioxidants. This term covers exogenous antioxidant substances entering the body with food and endogenous antioxidants regulating redox balance by chemical buffering or enzyme-catalysed free-radical detoxification. If protective systems are not capable to cope with the influence of free radicals the pathological condition termed oxidative stress develops an important part of the pathogenesis of various disorders. Treatment or prevention of such disorders requires administration of additional antioxidant substances contained in food, dietary supplements or even pharmaceuticals. Using high-performance liquid chromatographic analysis extracts of various sea cucumbers were shown to contain phenolic compounds such as chlorogenic acid, pyrogallol, rutin, coumaric acid, catechin, as well as ascorbic acid as potential antioxidants. These extracts were tested under in vitro conditions and results confirmed their high scavenging activity for nitric oxide radical, moderate iron-chelating activity, and inhibitory influence on lipid peroxidation processes. In animal studies rats were administered extracts of sea cucumbers that lead to increased superoxide dismutase and glutathione peroxidase activities. The use of hepatotoxic thioacetamide in combination with holothurian extracts resulted in lowered serum direct bilirubin level, activity of alanine and aspartate aminotransferases, concentrations of hepatic malondialdehyde and hydroxyproline as well as increased antioxidant enzyme activities [118]. Oral administration of Holothuria atra extract exerted hepatoprotective and antioxidant effects in rats with 7,12-dimethylbenzanthracene-induced hepatorenal dysfunction, namely, significantly diminished the level of the kidney dysfunction markers, serum creatinine, urea and uric acid, and normalized levels of malondialdehyde, reduced glutathione, glutathione-*S*-transferase, superoxide dismutase, and catalase in the liver tissue [119]. Similarly, Holothuria arenicola extract given to mice with liver disorder induced by bile duct ligation through gastric gavage normalized the antioxidant enzyme, glutathione-S-transferase, superoxide dismutase and catalase activities [120]. Several other sea cucumbers like *Holothuria arguinensis* from the North-Eastern Atlantic and *Holothuria atra* from the coast of Red Sea also were shown to have high antioxidant potential [121,122].

Obviously, in addition to phenolic compounds, other compounds can possibly have a role in the antioxidant activity in holothurians. Sea cucumber *Cucumaria frondosa*, mentioned before as a source of very potent triterpene glycosides, contains a complex of EPA-enriched PL (eicosapentaenoic acid-enriched phospholipids) that markedly reduced oxidative damage in the rat adrenal pheochromocytoma cell line PC12 induced by hydrogen peroxide (H_2_O_2_) and tert-butylhydroperoxide (*t*-BHP). Pretreatment of PC12 cells with EPA-enriched PL reduced damage and increased the survival rate in a dose-dependent manner. EPA-enriched PL also reduced lactate dehydrogenase leakage, and increased intracellular total antioxidant capacity of the cells and activity of superoxide dismutase, an important part of the cellular oxidation system because it can catalyze disproportionation reactions and clear free radicals. At the same time, pretreatment with EPA-enriched PL was found to down-regulate level of Bcl-2 mRNA and up-regulate Bax, Caspase-9, and Caspase-3 mRNA expression induced by H_2_O_2_ or t-BHP [7]. These results indicate that EPA-enriched PL from *Cucumaria frondosa* inhibit mitochondria-dependent pathways of apoptosis leading to substantial antioxidant effects.

Efficacy of lipid compounds from sea cucumbers was confirmed in other studies. Two lysophospholipids, lysoplatelet activating factor and lysophosphatidylcholine, were extracted from *Holothuria atra*. Pretreatment of the macrophage cell line J774A.1 with these lipid fractions reduced the rate of H_2_O_2_-induced apoptosis. At the same time oxygen radical absorbance capacity assay showed high antioxidant capacity for these lipids [123].

Besides lipid compounds, other active substances from sea cucumber possess marked antioxidant properties. Body walls of *Holothuria leucospilota* contain several saponins, which were shown in different in vitro systems to exert moderate antioxidant effects. For example, saponins showed DPPH (1,1-diphenyl-2-picrylhydrazyl) radical scavenging activity, ABTS (azino-bis-3-ethylbenzothiazoline-6-sulfonic acid) radical scavenging activity, ferric reducing power, and total antioxidant capacity. The isolated saponin complex also reduced viability of the human breast cancer cell line MCF7 in a dose-dependent manner for concentrations varying from 2.0 to 10.0 μg/mL and exerted pro-apoptotic activity via an increase of the Bax expression and decrease of the Bcl2 expression [124].

In addition to antitumor activity sea cucumber cerebrosides exert significant antioxidant effects. Sea cucumber *Acaudina molpadioides* cerebrosides with the following structural parameters: glycosyl group was glucose, the long-chain base only contained d17:1, the amide-linked fatty acid units consisted of C18:0 h, C22:1 h, C23:1 h, and C24:1 h. These cerebrosides significantly reduced lactate dehydrogenase leakage in PC12 cells and like previous lipid compounds restored damage caused by H_2_O_2_ or *t*-BHP. At the same time, decreased accumulation of intracellular ROS and increased activity of superoxide dismutase was noted after treatment with these saponins. Antioxidant effects of cerebrosides are likely linked to strengthening cell membranes under oxidative stress and enhanced activity of cellular antioxidant system components. Another possible mechanism for protective effects of holothurian cerebrosides may be increased gene expression of Bcl-2 and decreased gene expression of cytochrome C, caspases 3 and 9 as shown in an experiment with oxidative stress induced by H_2_O_2_ or t-BHP treatment [125]. Results of the in vivo studies showed that cerebrosides significantly decreased the levels of malondialdehyde, 8-oxo-7,8-dihydro-2′-deoxyguanosine, 8-hydroxy-2′-deoxyguanosine, and nitric oxide in demented mice with enhanced activity of superoxide dismutase [126]. Due to the variety of antioxidant mechanisms demonstrated, sea cucumber cerebrosides may be considered a potential pharmaceutical agent for the treatment of oxidative stress in different organs as well as in the central nervous system.

As with other marine edible invertebrates, sea cucumbers are a plentiful source of biologically active peptides that have a broad range of beneficial effects including antioxidant, antimicrobial, antiviral, antitumor, immunomodulatory, antihypertensive, anti-diabetic, analgesic, and neuroprotective. These active peptide molecules are promising prototypes for future development of the effective drugs [127]. 

Usually peptides with antioxidant properties are obtained via enzymatic hydrolysis of large marine proteins and typically have a low number of residues—from 2 to 20 [8]. They have a certain amount of hydrophobic and aromatic amino acids in a specific sequence. Antioxidant capacities of these peptides strictly correlate with the ability of their amino acid units to donate protons to free radical particles, chelate metal ions, or trap lipid peroxyl radicals [128]. Preparation of antioxidant peptides from marine invertebrates through protein hydrolysis is a relatively simple process because commercially available animal, plant, or microbial proteases such as, pepsin, trypsin, α-chymotrypsin, papain, alcalase, and neutrase are readily available.

It was discovered several decades ago, that collagen an essential component of connective tissue makes up 70% of all proteins in the body of sea cucumbers. For the ABTS assay peptide hydrolysates were obtained using trypsin hydrolysis of Stichopus vastus integument collagen and had a molecular mass ranging from 5 to 25 kDa. These peptides were able to quench ABTS radicals up to 71.3% [129]. Efficacy of low molecular peptides was shown by the antioxidant activity of hydrolysates and ultrafiltrate peptide fractions obtained from the boiled sea cucumber *Isostichopus badionotus* after simulated gastrointestinal digestion (pepsin + corolase PP). It was found out that oligopeptides with molecular weight lower than 3 kDa exerted 5 times higher free radical scavenging activity in comparison to peptides heavier than 3 kDa. Iron chelating potential of oligopeptides was 1.8 times higher [130]. These results obviously show that small peptides are generally the source of observed antioxidant effects.

Sea cucumber *A. japonicus* was also used as a source of active proteins, which were isolated and then hydrolyzed by pepsin, trypsin, papain, acid protease and neutral protease yielding five different peptide fractions. These fractions were evaluated for their antioxidant activity using scavenging hydroxyl radical (^−^OH) and superoxide anion (O_2_^−^). It was demonstrated that of the five tested fractions trypsin peptides had the maximum yield with highest antioxidative capacities. A pure trypsin peptide (TP2b-1) fraction was isolated. It was composed of the amino acid sequence as follows: Gly-Pro-Glu-Pro-Thr-Gly-Pro-Thr-Gly-Ala-Pro-Gln-Trp-Leu-Arg. The molecular weight of this fraction was about 1563 Da. The results were impressive as this type of peptide exerted antioxidant activity with IC_50_ 0.217 ± 0.008 mg/mL (138.9 μM) for ^−^OH and 0.553 ± 0.06 mg/mL (353.9 μM) for O_2_^−^. Artificial antioxidant compound butylated hydroxytoluene used as a positive control was more effective with IC_50_ values 8.75 mg/mL (39.7 μM) and 45.9 mg/mL (208.3 μM) for -OH and O_2_^−^ respectively. Although the purified peptide from this sea cucumber possesses less pronounced free-radical scavenging activity, it can be considered as a possible source to develop new antioxidants. The authors of this study concluded that taking into account the sufficiently high antioxidative potential of this holothurian peptide and the ratio between its hydrophilic and lipophilic forces, such a peptide may find new application as a food additive or functional food, and maybe even as a dietary nutrient and even a drug [131].

## 6. Angiotensin-Converting Enzyme Inhibitors

Angiotensin converting enzyme (ACE) is a key component of the complicated system for blood pressure regulation. ACE promotes conversion of inactive angiotensin I into the active vasoconstrictor compound angiotensin II via renin-angiotensin and the kinin-kallikrein systems. At the same time ACE contributes to inactivation of a specific vasodilator, bradykinin. Nowadays, ACE is considered the most effective biotarget in the treatment of blood pressure disorders. Besides numerous synthetic compounds there are plenty of natural sources which, can provide more effective and safe novel ACE inhibitors. Bioactive peptides from marine species are among them.

Peptide hydrolysates of proteins obtained from sea cucumber *Actinopyga lecanora* were prepared using several enzymes including alcalase, papain, bromelain, flavourzyme, pepsin, and trypsin. These hydrolysates demonstrated significant ACE inhibiting effects along with antioxidant activity. Alcalase hydrolysates were shown to have highest ACE inhibitory activity (69.8%) after 8 h of hydrolysis [132,133].

Sea cucumber *Stichopus horrens* was hydrolyzed using several enzymes. The outcomes showed that alcalase hydrolysate has the highest amount of protein (39.8%) followed by flavourzyme hydrolysate (32.7%). As expected alcalase hydrolysate showed the highest inhibitory activity regarding ACE with IC_50_ value of 0.41 mg/mL. Other hydrolysates obtained by this experiment also were found to have ACE inhibiting activity, but it was significantly lower. Flavourzyme hydrolysate had IC_50_ value 2.24 mg/mL, trypsin hydrolysate had IC_50_ value 2.28 mg/mL, papain hydrolysate had IC_50_ value 2.48 mg/mL, bromelain hydrolysate had IC_50_ value 4.21 mg/mL, and protamex hydrolysate had IC_50_ value of 6.38 mg/mL. It was noted that hydrolysis of the initial protein with alcalase led to the most substantial reduction in molecular weight making protein intensity very high (6 to 20 kDa). Hydrophobic and positively charged amino acids at the C-terminal are known to provide inhibitory properties to the peptide. Among all hydrolysates obtained in this study flavourzyme degraded sample A was shown to have the highest hydrophobic content. Alcalase and trypsin hydrolysates have fewer hydrophobic units. Positively charged amino acids were found in all the hydrolysates samples but did not meet significant IC_50_ values after testing for ACE inhibiting activity. The lowest IC_50_ was noted for alcalase hydrolysate sample with flavourzyme, trypsin, and bromelain hydrolysates following. These results suggest that the sequence of amino acids plays a more important role in ACE inhibiting activity than the total amount of hydrophobic amino acids [134].

Therefore, bioactive peptide hydrolysates from various sea cucumbers may be used as ingredients in manufacturing functional foods, supplements and pharmaceuticals reducing the risk of heart disease in hypertensive persons [135].

## 7. Immunity Stimulating Agents

Many triterpene glycosides isolated from different sea cucumber species were shown in both in vivo and in vitro experiments to be effective inductors of the cellular immunity [136,137,138]. Frondoside A and cucumarioside A2-2 from *C.f. japonica* [136,138] were used for treatment of peritoneal mouse macrophages in vitro in doses 0.1 and 0.02 μg/mL, respectively, and this resulted in the marked increase of lysosomal activity by 20–30%. The cucumariosides I2, B2 and A5 isolated from sea cucumber *Eupentacta fraudatrix* used in doses 1–5 μg/mL similarly increased lysosomal activity by 15–16%. Immunostimulating activity of these compounds depends on structural peculiarities. Even slight changes of triterpene glycoside structural characteristics resulted in dramatic changes in their effects. Immunomodulatory activity of sea cucumber glycosides depends on both aglycone and carbohydrate chain structures [139].

An acidic mucopolysaccharide was isolated from the body wall of sea cucumber *Stichopus japonicus*. It was composed of galactosamine, hexuronic acid, fucose and sulfate with a ratio of 1:1:1:4 and possessed numerous pharmacological properties including immune stimulating activity. Investigation of the effects of this acidic mucopolysaccharide in rats with diethylnitrosamine-induced hepatocellular carcinoma showed that oral administration of this compound for 5 days resulted in reduced number and mean volume of tumor nodes, decreased serum α-fetoprotein level, proliferated cell nuclear antigen expression in liver, and increased p21 expression. Furthermore, administration of acidic mucopolysaccharide led to a significantly decreased level of TNF-α and increased level of IL-2 in rat serum. It also contributed to improved function of spleen and thymus, enhanced activity of macrophage phagocytosis as well as NK cell-mediated tumoricidal activity. After a 5-day administration of the mucopolysaccharide a marked recovery of CD3+ and CD4+ T lymphocyte levels was noted and CD4+/CD8+ T cell ratio was normalized depending on the dose used [140]. The growth mechanism of hepatocellular carcinoma affected by mucopolysaccharide from sea cucumber *Stichopus japonicus* is very likely related to stimulation of immune organs and cell proliferation in rats resulting in enhanced cellular immunity pathways. Such influence is very important because enhanced immune activity in oncological patients has been considered a possible means for inhibiting of tumor growth. Generally, immune functional status and occurrence and progression of malignant tumors are closely related in patients.

Sea cucumbers as food ingredients improve systematic and mucosal immunity. It is interesting that immunomodulating effects are clearly seen in patients with immune deficiency disorders and they are not noted in healthy persons. In experimental studies with mice with cyclophosphamide-suppressed immunity, dietary *Apostichopus japonicus* was administered orally as lyophilized powder at different doses up to 512 mg/kg daily which is equivalent to half a whole sea cucumber prepared in an appropriate way. The results proved that sea cucumber intake enhanced respiratory and intestinal mucosal immunity in a dose-dependent manner. After cyclophosphamide injections that seriously damaged thymus and spleen in a test group, the thymus/bodyweight and the spleen/bodyweight indices in mice given 512 mg/kg of *A. japonicus* were significantly higher than in untreated animals. Such results suggest that dietary consumption of *A. japonicus* would stimulate an immune system impaired by chemotherapeutic treatment. Mechanisms of these effects are complicated and related to increased lysozyme expression, increased levels of secretory immunoglobulin A in respiratory and intestinal mucous membranes, and enhanced expression of polymeric immunoglobulin receptor in respiratory tract and intestinal organs [141]. Unfortunately, today it is not yet clear what compounds contained in sea cucumber walls may be responsible for these observed effects.

## 8. Antihyperlipidemic Agents

Nowadays, lipid metabolism imbalance is considered an important risk factor for atherosclerosis, cardiovascular diseases, and obesity which remain the most frequent causes of death worldwide. Thus, the problem of pharmacological regulation of lipid metabolism is very important. Conventional therapeutic agents purposed to correct elevated blood lipid level are generally cholesterol lowering drugs such as statins and fibrates. Although effective, these synthetic drugs cause a variety of adverse effects. Therefore, research interest is focused on new biologically active compounds from natural sources reducing blood lipid levels with a low risk of adverse reactions.

Several compounds with such activity are found in marine sources. Polysaccharides from sea cucumber *Apostichopus japonicus* were depolymerized using protease hydrolysis. The final polymer with an average molecular weight 36 kDa was composed of several units such as glucosamine, galactosamine, glucuronic acid, mannose, glucose, galactose and fucose. In animal studies this polysaccharide was given to hyperlipidemic male Wistar rats at a dose of 400 mg/kg daily resulting in significantly decreased levels of plasma total cholesterol, triglyceride, and low density lipoprotein cholesterol by 17.23%, 20.78% and 31.18%, respectively. At the same time a marked increase of high density lipoprotein cholesterol level by 27.3% compared to the hyperlipidemic group was noted [142].

Sea cucumber *Isostichopus badionotus* was lyophilized, cooked in water and lyophilized, or oven dried and tested in rats as a dietary component. Rats with high blood cholesterol level showed significant improvement after regular consumption of this sea cucumber. Noticeably decreased serum levels of cholesterol, low density lipoproteins, and triglycerides were followed by beneficial atherogenic index values. Also, concentrations of total lipids, triglycerides and cholesterol in the liver were reduced as well [143]. Saponins obtained from sea cucumber *Pearsonothuria graeffei* and then added to animal food in amount of 0.03% to 0.05% were shown to lower total lipid concentration in blood and liver, decrease serum triglyceride and total cholesterol levels and alleviate orotate-induced hepatic steatosis. Administration of saponins also led to inhibited activity of hepatic lipogenic enzymes such as fatty acid synthase, malic enzyme, and glucose-6-phosphate dehydrogenase and decreased expression of fatty acid synthase, malic enzyme, glucose-6-phosphate dehydrogenase, and sterol-regulatory element binding protein (SREBP-1c) genes. Furthermore, sea cucumber saponins increased activity of carnitine palmitoyl transferase in liver and up-regulated hepatic peroxisome proliferator-activated receptor (PPARa), its target gene, and expression of acyl-CoA oxidase mRNA [144]. Such detailed results suggest that the lipid-lowering activity mechanism of sea cucumber saponins is closely linked to the enhancement of β-oxidation inducing PPARa activation and inhibition of SREBP-1c-mediated lipogenesis. It was stated that sea cucumber saponins could ameliorate obesity, hepatic steatosis, and glucose intolerance in vulnerable people. It was confirmed by in vivo studies that a dietary marine saponin extract added to mouse food in the amount 0.1 to 0.3% caused weight-loss with substantially decreased adipose tissue in both visceral and subcutaneous depots. It also significantly reduced accumulation of triglyceride and total cholesterol in the liver, and decreased blood levels of glucose and insulin indicating that dietary saponins can enhance sensitivity of receptors to insulin. Also, this marine saponin extract increased adiponectin production preventing adipokine imbalance, and decreased level of tumor necrosis factor alpha induced by the high-fat diet [145].

A saponin fraction isolated from *Cucumaria frondosa* in animal studies with rats exerted cholesterol- and triglyceride-lowering effects by significantly suppressing lipid absorption in the intestine and accelerated fecal neutral sterol excretion. It was also shown to inhibit pancreatic lipase activity under in vitro conditions [146]. Similarly, saponin extracts and echinoside A extracted from sea cucumber *Pearsonothuria graeffei* possess anti-obesity properties due to effective inhibition of pancreatic lipase activity and up-regulation of LXR-β signaling [11]. Taking into account all these data, it may be deduced that lipid-lowering mechanisms of sea cucumber saponins are related to inhibition of the crucial enzymes involved in lipid metabolism.

A novel promising cerebroside was isolated from sea cucumber *Acaudina molpadioides* composed of four saturated and monounsaturated α-hydroxy fatty acids, a dihydroxy sphingoid base with one double bond (d17:1), and glucose. This cerebroside was administered to rats with non-alcoholic fatty liver disease (NAFLD) induced by orotic acid as a diet supplement in concentrations 0.006% and 0.03% and induced significantly decreased triglyceride and total cholesterol concentrations in liver. The higher doses of cerebroside contributed to a more pronounced lipid lowering activity in comparison to the positive control group given orotic acid diet [147]. The lipid lowering mechanism of dietary cerebroside was related to inhibition of liver stearoyl CoA desaturase (SCD) activity and mRNA expression. SCD-1 expression was suppressed by 37.5% in the group of rats given 0.006% of cerebroside in the food and by 61.8% in animals given food containing 0.03% of this compound in comparison with the positive control. The desaturation index commonly used for measuring of SCD-1 activity was decreased by 43.7% and 64.2% in animals given a diet containing 0.006% and 0.03% of this cerebroside, respectively, indicating inhibition of fatty acid desaturation [147]. The beneficial effects of this cerebroside isolated from *A. molpadioide* in animals with NAFLD were inhibiting SCD-1 activity and suppressing synthesis of monounsaturated fatty acids in rat hepatocytes.

FuCS was isolated from the sea cucumber Acaudina molpadioides and its effects on adipogenesis were evaluated in vitro as well as in high fat and high sucrose diet (HFSD) mice. Structural characteristics of this biopolymer were as follows: average molecular weight 21.53 kDa, sulfate content 27.81%, molar ratio of glucuronic acid, galactosamine and fucose is 1:1.14:1.55. The interesting results obtained indicated that FuCS inhibits differentiation of 3T3-L1 preadipocytes at the early and mid stages of differentiation and does not affect them at later stages in comparison to the control. The triglyceride level was 31.76%, 19.76% and 13.85% lower, respectively, after treatment with FuCS at the early, mid, and late stages. FuCS enhances expression of such Wnt/b-catenin related factors as Wnt10b, b-catenin, Fz and LRP resulting in a greater amount of cytoplasmic b-catenin translocated into the nucleus inhibiting the expression of PPARg and C/EBPa, and also expressions of SREBP-1c with transcriptional product. These data suggest that FuCS exerts significant anti-adipogenic effects related to activation of the Wnt/b-catenin pathway and down-regulation of SREBP-1c expression. Further studies showed FuCS given to mice significantly decreased protein expression of positive regulators of adipogenesis: SREBP-1c, PPARg and C/EBPa compared to the HFSD-fed animals. At the same time FuCS was shown to increase expression of b-catenin, a negative regulator in adipogenesis in adipose tissue, and decreased expression of SREBP-1c, PPARg and C/EBPa, positive regulators of adipogenesis [148].

FuCSs from other species of sea cucumbers such as, for example, *Isostichopus badionotus* and *Pearsonothuria graeffei* also possess lipid lowering activity. In experiments they lowered blood cholesterol, low density lipoprotein levels and the atherogenic index in high-fat high-fructose diet-fed C57BL/6J mice. Certain structural patterns provide potency to the lipid lowering activity of marine glycosaminoglycan. For instance, glycosaminoglycan with 3,4-*O*-disulfation fucose branches exert more pronounced lowering of blood lipid level than glycosaminoglycan with 2,4-*O*-disulfation [149]. These data suggest that FuCS from various sea cucumber species maybe successfully used in the prevention and treatment of obesity, atherosclerosis and their complications. Further studies should be focused on exploration of relationships between structural characteristics of biopolymers and their lipid lowering activity and would result in active compounds that can be used as drug prototypes or functional food constituents.

## 9. Anti-Diabetic and Glucose Lowering Activity

High blood glucose levels and associated accelerated lipid peroxidation involved in the pathogenesis of diabetes and its complications are caused by low activity of blood insulin due to insulin resistance or impaired insulin secretion in the pancreas leading to clinical manifestations of diabetes mellitus. Despite a wide variety of drugs purposed for treatment of diabetes, there is still no effective therapy reducing high glucose levels, providing cells with sufficient glucose supply, and preventing severe hyperglycemic complications. Thus, discovery of new agents with hypoglycemic and antioxidant activities is very important.

Some compounds from sea cucumbers were shown to exert glucose lowering activity. For instance, a saponin fraction obtained from *Holothuria thomasi* exerts anti-diabetic effects. The aglycones of these saponins have methyl esters of octadecanoic acid whereas the aglycone moiety is a typical triterpenoid. Significant decrease of blood glucose levels and increase of blood insulin was noted in rats with streptozotocin-induced hyperglycemia and insulin deficiency after administration of the saponin fraction from the sea cucumber. Besides reduction of blood glucose levels, saponin administration also led to reduced α-amylase activity, and lowered concentration of adiponectin, pro-inflammatory cytokines interleukin-6 and tumor necrosis factor alpha in serum and l-malondialdehyde in liver. It was associated with an increased amount of glycogen stored in liver. Morphological studies revealed that rats given the saponin extract had less pronounced degenerative alterations in pancreas β-cells [150]. Some research studies have focused on the influence of saponins on various links in diabetic pathogenesis. A saponin extract from *Pearsonothuria graeffei* containing mainly holothurin A and echinoside A exerted marked α-glucosidase inhibitory activity. The saponins from *P. graeffei* are generally triterpene glycosides composed of a lanosterol-type aglycone with a saccharide moiety attached at the C-3 position. The saponin fraction was found to be a more effective inhibitor of yeast α-glucosidases activity (IC_50_ = 0.04 mg/mL) than the antidiabetic drug acarbose (IC_50_ = 1.06 mg/mL) in vitro. Animal studies did not confirm this promising data as the marine saponin fraction did not inhibit rat intestinal α-glucosidase as effectively (IC_50_ = 0.086 mg/mL) as acarbose did in vivo (IC_50_ = 0.02 mg/mL). Nevertheless, postprandial blood glucose levels shortly after oral administration of the saponin fraction via gastric gavage increased dramatically, probably, due to the induction of corticosterone secretion by adrenals [151]. At the same time long-term administration of the saponin fraction from another sea cucumber was shown to significantly improve glucose tolerance [145].

Sea cucumber FuCS, mentioned several times in this review due to its different pharmacological activities has also shown antidiabetic effects. This polysaccharide from *Cucumaria frondosa* with an average molecular weight 14.76 kDa mainly composed of glucuronic acid, galactosamine, and fucose (molar ratio of 1:1.5:1.16) and a sulfate 30.07% content was given to type 2 diabetic mice on a high-fat high-sucrose (HFSD) diet resulting in significantly improved insulin sensitivity in skeletal muscle, and reduced blood glucose and insulin levels. The complex biochemical influence of FuCS increases sensitivity of cells to insulin by reversing of HFSD-induced decreases in glucose transporter 4 (GLUT4) translocation to the plasma membrane and increased phosphorylation of phosphoinositide 3-kinase at p85, protein kinase B at Ser473, and Thr308 in skeletal muscle. This polysaccharide activates insulin signaling via increased mRNA expressions of key genes regulating glucose uptake and induction of phosphatidylinositol 3 kinase/protein kinase B signaling in skeletal muscles. Effects of FuCS administered to mice in a 80 mg/kg daily dose were very similar to rosiglitazone given daily at a 1 mg/kg dose [152]. It should be mentioned that rosiglitazone is considered a potent antidiabetic drug reducing blood glucose level and enhancing sensitivity of adipose tissue, skeletal muscle and liver tissue to insulin. So, FuCS may be used for therapeutic purposes. This suggestion is confirmed by experimental results in mice showing that combined administration of FuCS and rosiglitazone leads to decreased body weight gain, reduced liver and blood glucose levels, and increased insulin concentration and glycogen stored in liver. These two compounds administered together act like synergists strengthening the effects of each other. After administration of both FuCS and rosiglitazone there were noted normalized activities of hexokinase, pyruvate kinase, and glucose-6-phosphatase in hepatocytes as well as increased mRNA expression of insulin receptors, insulin receptor substrate 2, phosphatidylinosotol 3 kinase, protein kinase B, and glycogen synthase, and suppressed glycogen synthase kinase 3β m-RNA expression in liver [153]. Therefore, administration of FuCS has two main advantages: decreased blood glucose levels via promotion of glycogen synthesis in the liver and possibly reducing rosiglitazone dosage with an increase in insulin sensitivity.

Sulfated polysaccharide fucoidan also exhibited anti-hyperglycemic activities in insulin resistant C57BL/6J mice. The monosaccharides of the fucoidan sample used in a study were fucose, galactosamine, galactose and glucosamine with a ratio of 1:0.1:0.3 0.17 with a 29.31% sulfate content. Daily administration of 80 mg/kg of fucoidan showed several beneficial effects. It significantly reduced fasting blood glucose and insulin levels, and increased glucose and insulin tolerance in insulin-resistant mice. In addition, it helped to increase the mRNA expressions of insulin receptors, insulin receptor substrate 1, phosphatidylinositol 3 kinase, protein kinase B, and glucose transporter 4 [12]. These results clearly show that this sulfated polysaccharide from sea cucumbers exerts anti-hyperglycemic effects in insulin resistant mice via activating the PI3K/PKB pathway and GLUT4.

Other compounds from sea cucumbers have shown antidiabetic activity. Two unsaturated fatty acids with α-glucosidase inhibitory activity, 7(Z)-octadecenoic acid and 7(Z),10(Z)-octadecadienoic acid, were isolated from sea cucumber *Stichopus japonicus* gathered from the Eastern coastal area of the Korean peninsula. They were shown to have some antidiabetic effects such as inhibition of *Saccharomyces cerevisiae* α-glucosidase with IC_50_ values at 0.51 and 0.67 μg/mL, respectively, and *Bacillus stearothermophilus* α-glucosidase, with IC_50_ values at 0.49 and 0.60 μg/mL. Inhibition constants for inhibitor binding with free enzyme, K_I_, determined by the Dixon plot and with enzyme–substrate complex, K_IS_, determined by secondary replots of these compounds against *S. cerevisiase* α-glucosidase were 0.44 and 0.22 μg/mL for first acid and 0.39 and 0.13 μg/mL for second one. So, 7(Z),10(Z)-octadecadienoic acid was more effective than 7(Z)-octadecenoic acid [154].

Eicosapentaenoic acid-enriched phosphatidylcholine was isolated from the sea cucumber *Cucumaria frondosa* and its antidiabetic effects were investigated on streptozotocin-induced hyperglycemia in rats given 25 and 75 mg/kg body weight of this compound through gastric gavage. Phosphatidylcholine from *C. frondosa* significantly reduced the blood glucose concentration and enhanced insulin secretion by restoring β-cells and glycogen synthesis in diabetic rats. It promoted the expressions of genes for insulin receptor, insulin receptor substrate-1, phosphoinositide 3-kinase, protein kinase B, and glucose transporter 4 in skeletal muscles and enhanced phosphorylation of Tyr-IR-b, p85-PI3K, and Ser473-PKB [155]. The mechanism for hypoglycemic influence of phosphatidylcholine from sea cucumbers was also investigated. Its up-regulating effects on PI3K/PKB signal pathway mediated by insulin were noted. Phosphatidylcholine may be considered a prototype for potent pharmaceuticals as well as a constituent of functional food intended for adjunctive therapy for diabetes mellitus. But, phosphatidylcholine has yet to be validated in the human clinical trials. 

## 10. Final Conclusions

Sea cucumbers are marine benthic invertebrates, dwelling in the seas across the world, in both temperate and tropical oceans, and from the intertidal zone to the deep sea. In most countries in Asia and Oceania, sea cucumbers are commonly used as a food or processed components for nutritional purposes. Their common names are bêche-de-mer, or *gamat*, or *trepang* (countries in Indian ocean and Malaysia), *teripang* (Indonesia), *namako* (Japan), *plingkao* (Thailand), *haishen*, or *hai-som* (China). In international trade, the term *sandfish* is also frequently used. Sea cucumbers or holothurians include about 14,000 known species and more than 70 of them are used throughout the world. But only about a dozen of sea cucumbers have commercial value [57,156]. The main customer countries for sea cucumbers are China and Hong Kong, South Korea, Singapore, Malaysia and Japan. In addition, significant amounts of sea cucumbers are being exported to some areas of United States and Australia [157].

For a long time, sea cucumbers have been used for nutritional purposes and, due to their efficacy, in folk medicine in Asian and Far Eastern countries as a traditional remedy for high blood pressure, asthma, rheumatism, skin cuts and burns, erectile dysfunction, and constipation [158]. They are barely known in Western countries. However, within the last three to four decades, sea cucumbers have become popular among biomedical researchers due to their nutritional values as well as their potential health benefits and possibility for therapeutic purposes. Several pharmacological activities of sea cucumbers namely anticoagulant and antiplatelet, antitumor, antihypertensive, antioxidant and antimicrobial have been described in recent science papers. These effects are likely due to the presence of biologically active compounds.

For industrial use, sea cucumbers have two potential values. First, they produce pharmacologically active compounds that can be of interest to pharmaceutical companies as potential drug prototypes. Second, they are already in use as a food product. Biologically active compounds of sea cucumbers can be divided into two groups: the first group may include unique compounds typical of holothurians with pharmacological activities that can become the basis for the development of novel pharmaceuticals (sulfated fucans, fucosylated chondroitin sulfates, triterpene glycosides, and sea cucumber cerebrosides). These substances may be pharmacologically potent. Te second group contains a wide array of bioactives, cerebrosides, chondroitin sulfates, fatty acids, glycosphingolipids, minerals (calcium, iron, magnesium, and zinc), lectins, peptides, phenolics, phospholipids, polysaccharides, saponins, sterols, and vitamins (niacin, retinol, riboflavin, and thiamine), and demonstrates a number of therapeutic effects leading to prospective use as functional foods and nutraceuticals. These compounds are not specific to holothurians only, but the nutritional value of sea cucumbers provides physiological benefits due to the wide spectrum of biological activities and similarity of biological effects of various classes of compounds to form additive and even synergistic effects. A typical example is a group of substances with antioxidant activity such as bioactive peptides, dietary cerebrosides, phospholipids and polyunsaturated fatty acids which, in combination, can reproduce an additive or synergistic antioxidant effect. So, the combination of active compounds from sea cucumbers in modern functional foods offers practical approaches to prevent various chronic diseases and maintain optimal human health due to these natural products with physiological benefits [159].

One of the main tasks of pharmacology and pharmacy is the search for sources of novel pharmaceutical molecules. From this point of view, the most promising compounds may be sulfated fucans, fucosylated chondroitin sulfates, triterpene glycosides, and cerebrosides found in different species of sea cucumbers. Each new compound isolated from a sea cucumber can be considered as a new pharmaceutical substance with a unique structure and, hence, potentially new pharmacological activities. 

Sulfated fucans from the sea cucumbers belong to a group of well-studied sulfated polysaccharides isolated from non-mammals but exerting biological activity in mammalian systems. The main area for their usage is treatment of thromboembolic disorders requiring regular use of anticoagulant therapy. FuCS as well as sulfated fucans possess heparin-like anticoagulant and antithrombotic activities. In contrast to typical anticoagulant pharmaceuticals like non-fractioned heparin and low molecular heparins, sulfated fucans and FuCSs from sea cucumbers exert less side effects, and their usage is associated with a lower risk of complications such as bleeding and thrombocytopenia. Moreover, the occurrence of side effects is less with lower molecular weight sea cucumber polysaccharides. In addition, FuCSs have antimalaria properties.

Triterpenoids, including triterpene glycosides, are the most abundant group of secondary metabolites found in marine sources including sea cucumbers. This review demonstrates sea cucumber triterpene glycosides are potential drug prototypes for the development of novel pharmaceuticals with antitumor activity. A range of triterpene glycosides, holothurin B, marmoratoside A, impatienside A, and bivittoside D also exert powerful antifungal effects which sometimes are close to modern antifungal pharmaceuticals, and are considered potentially novel antifungal drugs. Regarding antioxidant, immune stimulating and glucose-lowering activity demonstrated by triterpene glycosides from sea cucumbers, our opinion is that presently, there is not sufficient grounds to consider them as potential prototypes for antioxidant, immune stimulating and glucose-lowering pharmaceuticals. At the same time, the high antileishmanial activity of holothurin B raise some practical interest but some additional comparison studies with other glycosides and pharmaceuticals are required.

Regarding the sea cucumber cerebrosides, they deserve attention because of their potential capacity to protect the gut from inflammation and tumors. It should be mentioned that such specific activity is typical of cerebrosides themselves as well as of their sphingoid bases. Currently, data is insufficient and further studies are needed to focus on the spectrum of cerebroside activity as well as their activity against various cancers. At the same time, such pharmacological effects of holothurian cerebrosides already described in research papers, such as antioxidant and lipid-lowering, make it possible to consider these compounds as potential nutrients and functional food components.

Thus, our knowledge about the effects of bioactive compounds isolated from the sea cucumbers makes it possible to consider these marine invertebrates as a promising source of substances for development of pharmaceuticals with at least anticoagulant, antithrombotic, antitumor, antifungal and antileishmanial activities. It is important to emphasize that the use of sea cucumbers as pharmaceutical raw materials would avoid the risk of contamination with pathogens like prions and viruses during isolation of pharmaceutical substances [160].

Despite the common opinion that natural products are an inexhaustible source of novel therapeutic agents, the discovery process of the new bioactive metabolites from natural sources is limited due to the chemical complexity of biological matrices containing these molecules. This fully applies to bioactive components of sea cucumbers. Moreover, the vast diversity of active compounds is quite impressive and includes dozens of types of polysaccharides, cerebrosides, sphingoid bases and hundreds of types of triterpeniods posing a serious problem for researchers to select the most promising lead molecules. Isolation and purification of the separated components requires many resources, in particular, financial. According to the principles of drug discovery, molecules must have high potential value. Thus, these molecules must be structurally novel and possess pharmacological activity [3,161,162]. Information provided in this present review demonstrates the pharmacological activity of components isolated from sea cucumbers. We hope that the data will create suggestions for further development strategies of new effective and safe pharmaceuticals.

## Figures and Tables

**Table 1:** Taxonomy of sea cucumbers containing bioactive compounds with experimentally confirmed pharmacological activity (Holothurian classification is presented in accordance with World Register of Marine Species).

Species *	Bioactive Compounds
Order Dendrochirotida Family Cucumariidae	
*Cercodemas anceps* (*Colochirus anceps*)	Triterpene glycoside: colochiroside A
*Cucumaria frondosa*	Polysaccharides: fucosylated chondroitin sulfate; Triterpene glycosides: frondosides A, A1, A6, 24-dehydrofrondoside A6; Cerebrosides; Phospholipids
*Cucumaria frondosa japonica* (*Cucumaria japonica*)	Triterpene glycosides: cucumariosides A2-2, A4-2
*Colochirus quadrangularis* (*Pentacta quadrangularis*)	Triterpene glycosides: pentactasides I, II, III, philinopsides A, B, E,
*Pseudocolochirus violaceus*	Triterpene glycosides: violaceusides A, B
*Mensamaria intercedens*	Triterpene glycoside: intercedensides A, B, C
**Family Phyllophoridae**	
*Massinium magnum*	Polysaccharides: fucosylated chondroitin sulfate
**Family Psolidae**	
*Psolus patagonicus*	Triterpene glycoside: patagonicoside A
**Family Sclerodactylidae**	
*Eupentacta fraudatrix*	Polysaccharides: fucosylated chondroitin sulfate; Triterpene glycosides: cucumariosides A5, B2, I2
**Order Holothuriida** **Family Holothuriidae**	
*Actinopyga agassizi*	Holothurin A
*Actinopyga echinites*	Ethanol extract
*Actinopyga lecanora*	Peptide hydrolysates, Triterpenes Holothurin A, B, methanol extract
*Actinopyga miliaris*	Ethanol extract
*Bohadschia argus*	Triterpene glycosides: argusides A, B, C, D, E; Galactocerebroside
*Bohadschia marmorata*	Triterpene glycosides: marmoratoside A, impatienside A, 17α-hydroxy impatienside A, bivittoside D
*Holothuria arenicola*	Phenolic compounds
*Holothuria arguinensis*	Polyunsaturated fatty acids: arachidonic, eicosapentaenoic, docosahexaenoic acids; Amino acids
*Holothuria atra*	Phospholipids: lysoplatelet activating factor, lysophosphatidylcholine, ethanol extract
*Holothuria edulis*	Polysaccharides: sulfated fucan, fucosylated chondroitin sulfate
*Holothuria forskali*	Polysaccharides: fucosylated chondroitin sulfate
*Holothuria fuscocinerea*	Triterpene glycosides: fuscocinerosides A, B, C
*Holothuria grisea (Ludwigothuria grisea)*	Triterpene glycosides: 17-dehydroxyholothurinoside A, griseaside A
*Holothuria hilla*	Triterpene glycoside: hillaside C
*Holothuria impatiens*	Triterpene glycosides: impatienside A, 17α-hydroxyimpatienside A
*Holothuria leucospilota*	Polysaccharides: glycosaminoglycan; Saponin complex
*Holothuria mexicana*	Polysaccharides: fucosylated chondroitin sulfate
*Holothuria moebii*	Triterpene glycosides: moebioside A, holothurin A, holothurin B, 24-dehydroechinoside B
*Holothuria nobilis*	Polysaccharides: sulfated fucan, fucosylated chondroitin sulfate
*Holothuria scabra*	Triterpene glycosides: scabrasides B, D, echinoside A, 24-dehydroechinoside A, HS-1, holothurins A, A1, A3, A4, B, B4, fuscocineroside C
*Holothuria thomasi*	Saponin complex
*Pearsonothuria graeffei*	Triterpene glycosides: echinoside A, Ds-echinoside A, 24-dehydroechinoside A, holothurin A; Polysaccharides: fucosylated chondroitin sulfate; Cerebrosides
**Order Molpadiida** **Family Caudinidae**	
*Acaudina molpadioides*	Polysaccharide: fucosylated chondroitin sulfate; Cerebrosides
**Order Synallactida** **Family Stichopodidae**	
*Apostichopus japonicus (Stichopus japonicus)*	Polysaccharides: sulfated fucan, fucosylated chondroitin sulfate; Triterpene glycosides: holotoxins A, B, D, E, F, G, H; Cerebrosides; Fatty acids: 7(Z)-octadecenoic acid, 7(Z),10(Z)-octadecadienoic acid; Active proteins
*Isostichopus badionotus*	Polysaccharides: fucosylated chondroitin sulfate, sulfated fucan; Low molecular peptides
*Stichopus horrens (Stichopus variegates)*	Triterpene diglycosides: stichorrenosides A, B, C, D, stichoposides A, B, 3β-*O*-[β-d-xylopyranosyl-(1→2)-β-d-xylopyranosyl]-23S-acetoxyholost-7-ene, 3β-*O*-[β-d-xylopyranosyl-(1→2)-β-d-xylopyranosyl]-23S-hydroxyholost-7-en; Enzyme hydrolysates; Cerebrosides
*Stichopus vastus*	Collagen hydrolysates
*Thelenota ananas*	Polysaccharides: fucosylated chondroitin sulfate
*Thelenota anax*	Triterpene glycosides: stichoposide C, cucumarioside A2-2, cucumarioside A4-2

* Taxons in parentheses are synonyms.

**Table 2:** Comparative chemical composition and average molecular weight of sea cucumber polysaccharides.

Sample	Source	Chemical Composition (Molar Ratios)				Mn (kDa)	M_W_ (kDa)	M_W_/Mn	Reference
		GlcA	GalNAc	Fuc	Glc				
Sulfated fucan	*Holothuria edulis*	<0.01	<0.01	1.00	<0.01	517.90	615.50	1.19	[17]
Sulfated fucan	*Apostichopus japonicas*	<0.01	<0.01	1.00	<0.01	225.90	419.90	1.86	
Sulfated fucan	*Holothuria nobilis*	<0.01	<0.01	1.00	<0.01	289.40	475.80	1.64	
Fucosylated chondroitin sulfate	*Holothuria edulis*	1	1.28	0.82	<0.01	41.34	51.09	1.24	
Fucosylated chondroitin sulfate	*Apostichopus japonicas*	1	1.05	1.03	<0.01	46.76	56.82	1.22	
Fucosylated chondroitin sulfate	*Holothuria nobilis*	1	0.96	0.82	<0.01	42.46	55.32	1.30	
Neutral glucan	*Holothuria edulis*	<0.01	<0.01	<0.01	1.00	197.90	253.30	1.28	
Heparin (commercial drug)	Porcine intestine	ND	ND	ND	<0.01	20.23	26.26	1.30	
Neutral glucan	*Holothuria edulis*	<0.01	<0.01	<0.01	1.00	197.90	253.30	1.28	
Fucosylated chondroitin sulfate	*Isostichopus badionotus*	1.43	1	1.71	ND	94.78	109	1.15	[22]
Depolymerized fucosylated chondroitin sulfate		1.35	1	1.70	ND	4.60	7.4	1.61	
Depolymerized fucosylated chondroitin sulfate		1.32	1	1.72	ND	2.89	5.2	1.80	
Depolymerized fucosylated chondroitin sulfate		1.30	1	1.71	ND	2.38	4.3	1.80	
Low molecular weight heparin (commercial drug)	Porcine intestine					5.0	6.4	1.28	

GlcA: glucuronic acid; GalNAc: *N*-acetyl-β-d-galactosamin; Fuc: α-l-Fucose; Glc: d-glucose; Mn: number-average molecular mass; M_W_: weight-average molecular mass; M_W_/Mn: molecular weight distribution; ND: not determined.

**Table 3:** Comparative anticoagulant activities o sea cucumber polysaccharides and heparins.

Sample	Source	M_W_ (kDa)	APTT (IU/mg)	TT (IU/mg)	TP (IU/mg)	Reference
Sulfated fucan	*Holothuria edulis*	615.50	13	<1	<1	[17]
Sulfated fucan	*Apostichopus japonicas*	419.90	19	<1	<1	
Sulfated fucan	*Holothuria nobilis*	475.80	9	<1	<1	
Fucosylated chondroitin sulfate	*Holothuria edulis*	51.09	89	6	<1	
Fucosylated chondroitin sulfate	*Apostichopus japonicas*	56.82	116	7	<1	
Fucosylated chondroitin sulfate	*Holothuria nobilis*	55.32	59	4	<1	
Neutral glucan	*Holothuria edulis*	253.30	<1	<1	<1	
Heparin	Porcine intestine (commercial drug)	26.26	212	212	212	
Sulfated fucan	*Isostichopus badionotus*	450	9	6		[20]
Fucosylated chondroitin sulfate	*Isostichopus badionotus*	Not specified	183	157		
Heparin	Porcine intestine (commercial drug)		150	150		
Fucosylated chondroitin sulfate	*Isostichopus badionotus*	109	187	157		[22]
Depolymerized fucosylated chondroitin sulfate *	*Isostichopus badionotus*	7.4	103.8	34.3		
Depolymerized fucosylated chondroitin sulfate *	*Isostichopus badionotus*	5.2	60.5	<1		
Depolymerized fucosylated chondroitin sulfate *	*Isostichopus badionotus*	4.3	34.8	<1		
Low molecular weight heparin	Porcine intestine (commercial drug)	6.4	69	64		
Heparin	Porcine intestine (commercial drug)	18.6	212	212		

M_W_: molecular weight; APTT: activated partial thromboplastin time; TT: thrombin time; PT: prothrombin time. The anticoagulant activity is expressed as USP (United States Pharmacopeia) units/mg (IU/mg) using a parallel standard curve based on the International Heparin Standard (212 or 150 IU/mg). * The depolymerized fucosylated chondroitin sulfate samples were prepared using modified free-radical depolymerization induced by Cu^2+^ catalyzed Fenton system.

**Table 4:** Comparative in vitro antithrombotic activities of sea cucumber polysaccharides and heparins.

Thrombogenesis in the Artificial Blood Vessel						
Sample	Source	M_W_ (kDa)	Length of Thrombus (cm)	Weight of Thrombus (mg)		Reference
Normal blood			2.16 ± 0.52	110.47 ± 9.49		[20]
Sulfated fucan (0.5 mg/mL)	*Isostichopus badionotus*	450	2.02 ± 0.24	40.58 ± 8.05 ^a^		
Sulfated fucan (1.0 mg/mL)			2.05 ± 0.26	35.08 ± 5.10 ^a^		
Fucosylated chondroitin sulfate (0.3 mg/mL)		Not specified	1.80 ± 0.33	35.40 ± 7.15 ^a^		
Fucosylated chondroitin sulfate (0.5 mg/mL)			1.73 ± 0.42	32.73 ± 5.35 ^a^		
Heparin (Jiangsu Pharmacia, China) (0.3 mg/mL)			ND	ND		
**Antithrombotic Potency Values**						
			Factor Xa/Antithrombin *	Thrombin/Antithrombin *	Thrombin/HCII **	
			**IU/1 mg of sample**			[40]
Native fucosylated chondroitin sulfate	*Holothuria forskali*	120–140	0.40	0.56	120	
Depolymerized fucosylated chondroitin sulfate		6	0.46	<limit of detection	7.8	
Depolymerized fucosylated chondroitin sulfate		2.5	0.181	<limit of detection	0.65	
Heparin			203	196	206	
			**IC_50_ (μg/mL)**			[18]
Sulfated fucan	*Holothuria edulis*	616	>1000	78.7 ± 6.2	0.7 ± 0.02	
Sulfated fucan	*Holothuria grisea*	564	>1500	66.5 ± 11	0.5 ± 0.03	
Heparin			0.03 ± 0.003	0.015 ± 0.001	0.2 ± 0.01	
			**IC_50_ (μg/mL)**			[20]
Sulfated fucan	*Isostichopus badionotus*	450	0.53	0.32	2.55	
Fucosylatedchondroitin sulfate		Not specified	2.58	0.56	0.05	
Heparin			0.005	0.01	0.98	

ND: thrombus not detected; HCII: heparin cofactor II; IU: international unit; IC_50_: half maximal inhibitory concentration; * samples were tested against the 6th International Standard for unfractionated heparin (07/328); ** samples were tested against the 2nd International Standard for low molecular weight heparin (01/608). ^a^ *p* < 0.05, compared with normal blood.

**Table 5:** Cytotoxic effects of sea cucumber triterpene glycosides against various tumor cells.

Compounds	Type of Tumor Cell Lines	Activity Result, IC_50_	Reference
Arguside A	Human leukemia HL-60, human colon cancer HCT-116, human hepatocellular carcinoma BEL-7402, human stomach adenocarcinoma MKN45	0.14–4.42 μM	[61]
Arguside B	Human lung cancer A549, HCT-116, human hepatocellular carcinoma human breast cancer MCF-7	0.46–2.60 μg/mL	[62]
Arguside C	A549, HCT-116, HepG2, MCF-7	0.38–2.56 μg/mL	[62]
Arguside D	A549, HCT-116, HepG2, MCF-7	3.45–7.77 μg/mL	[63]
Arguside E	A549, HCT-116, HepG2, MCF-7	3.36–7.53 μg/mL	[63]
Bivittoside D	Colon carcinoma HT-29, HCT-116, human prostate cancer DU-145, MCF-7, human epidermoid carcinoma KB, A549, HepG2	0.37–2.46 μg/mL	[64]
Colochiroside A	Murine leukemia P-388, HL-60, A549, lung adenocarcinoma SPC-A4, stomach adenocarcinoma MKN28, gastric carcinoma SGC-7901	3.61 ± 0.55 μg/mL	[65]
Cucumarioside A2-2	HL-60, human leukemia THP-1, human leukemia NB-4, human leukemia K562	Effective concentration: ≥5 μM	[66]
Cucumarioside A4-2	HL-60, THP-1, NB-4, human leukemia K562	Effective concentration: ≥5 μM	[66]
Echinoside A	Human cervical cancer HeLa, HepG2, K562	1.25–1.61 μg/mL	[67]
	HepG2	2.70 μmol/L	[68]
Ds-echinoside A	HepG2,	2.65 μmol/L	[69]
24-Dehydroechinoside A	HepG2	3.14 μM	[70]
	HeLa, HepG2, K562	1.95–6.15 μg/mL	[67]
24-Dehydroechinoside B	Rat glioma C6, human glioma U-87-MG, human glioma U251, human glioma SHG-44	1.99–6.10 μM	[71]
Frondoside A	Human pancreatic cancer AsPC-1	Effective concentration: 4 μg/mL	[72]
	Human lung cancer LNM35, A549, human lung carcinoma NCI-H460-Luc2, human melanoma MDA-MB-435, human mammary adenocarcinoma MCF-7, HepG2	1.7–2.5 μM	[73]
	A549	0.6 μM	[74]
	HeLa, HepG2, K562	3.30–4.14 μg/mL	[67]
Frondoside A1	HeLa, HepG2, K562	1.91–2.21 μg/mL	[67]
Fuscocineroside A	HL-60, BEL-7402	0.58–0.88 μg/mL	[75]
Fuscocineroside B	HL-60, BEL-7402	0.58–0.88 μg/mL	[75]
Fuscocineroside C	HL-60, BEL-7402	0.58–0.88 μg/mL	[75]
Griseaside A	A549, HL-60, BEL-7402, human acute lymphoblastic leukemia Molt-4	0.427–2.60 μM	[76]
Hillaside C	A549, MCF-7, human lung carcinoma IA9, human clear cell carcinoma CAKI-1, human prostate adenocarcinoma PC-3, KB, nasopharyngeal cancer KB-VIN, HCT-8	0.15–3.20 μg/mL	[77]
Holothurin A	HL-60, BEL-7402	0.58–0.88 μg/mL	[75]
	HeLa, HepG2, K562	3.46–8.94 μg/mL	[67]
	C6, U87-MG, U251, SHG-44	0.99–4.03 μM	[71]
Holothurin A1	HepG2,	3.40 μM	[70]
Holothurin A1	HeLa, HepG2, K562	2.84–6.50 μg/mL	[67]
Holothurin A3	KB, HepG2	0.32–0.87 μg/mL	[78]
Holothurin A4	KB, HepG2	0.57–1,12 μg/mL	[78]
Holothurin B	HeLa, HepG2, K562	1.79–3.64 μg/mL	[67]
Holothurins B	C6, U87-MG, U251, SHG-44	1.39–8.64 μM	[71]
Holothurin B4	HeLa, HepG2, K562 IC_50_ = 2.71–3.55	2.71–3.55 μg/mL	[67]
Dehydroxyholothurinoside A	A549, HL-60, BEL-7402, Molt-4	0.245–0.97 μM	[76]
Impatienside A	HT-29, HCT-116, DU-145, MCF-7, KB, A549, HepG2	0.353–2.72 μg/mL	[64]
Intercedensides A, B, C	A549, MCF-7, IA9, CAKI-1, U-87-MG, PC-3, KB, KB-VIN, human skin melanoma SK-MEL-2, HCT-8	0.60–4.00 μg/mL	[79]
Moebioside A	C6, U87-MG, U251, SHG-44	1.22–4.39 μM	[71,80]
Patagonicoside A	Human hepatocarcinoma Hep3B, breast cancer MDA-MB-231, A549	0~80 μM	[81]
Pervicoside C	HCT-116 and A549	18.7–28.6 μg/mL	[64]
Pentactasides I, II, III	P-388, A549, MCF-7, MKN28, HCT-116, U87-MG	0.60–3.95 μM	[82]
Philinopside A	P-388, A549, MCF-7, MKN28, HCT-116, U87MG	0.60–3.95 μM	[82]
	BEL-7402, MCF-7, ovarian epitheloid carcinoma HO-8910, mouse Sarcoma-180	1.5–2.4 μM	[83]
Philinopside B	P-388, A549, MCF-7, MKN28, HCT-116, U87MG	0.60–3.95 μM	[82]
Philinopside E	MKN28, P-388, BEL-7402, HL-60, SPC-A4, A549, SGC7901, human ovarian carcinoma HO8901, human fetal lung fibroblasts W138, human epithelial carcinoma A431	0.75–3.50 μg/mL	[84]
	BEL-7402, SPC-A4, ovarian epitheloid carcinoma HO-8910	2.4–4.1 μM	[85]
Scabraside B	HeLa, HepG2, K562	4.44–11.85 μg/mL	[67]
Stichoposide C	HL-60, K562, NB-4, THP-1, colorectal cancer CT-26, HT-29, colon adenocercinoma SNU-C4	Effective concentrations: 0.3–1.5 μmol/L	[86]
Scabraside D	HeLa, HepG2, K562	3.33–10.06 μg/mL	[67]
Stichorrenosides A, B, C, D	KB, HepG2, MCF-7, human prostate adenocarcinoma LNCaP cells, SK-Mel-2	1.31–3.53 μM	[9]
Violaceusides I, II, III	MKN45, HCT-116	0.068–0.352 μM	[87]

**Table 6:** Cytotoxicity of sea cucumber cerebrosides against cancer cells.

Compounds	Source	Type of Tumor Cell Lines	Pharmacological Effect, Anticancer Mechanism	Reference
Sphingoid bases	*Stichopus horrens*	Human colon cancer cells Caco-2, DLD-1 and WiDr	Reduction of cell viability, induction of apoptosis, increasing caspase-3 activity	[96]
Glucocerebrosides, sphingoid bases	*Cucumaria frondosa*	Caco-2	Inhibition of cell proliferation	[99]
Glucocerebrosides	*Acaudina molpadioides*	Murine sarcoma cells S180	Induction of apoptosis	[100]
Sphingoid bases	Species not specified	Human hepatoma cells Hep-G2	Reduction of cell viability, induction of apoptosis, upregulation of death receptor-5, apoptosis inducer protein Bax, growth arrest and DNA-damage-inducible protein DNA-damage-inducible gene 45 and peroxisome proliferator-activated receptor-γ, downregulation of protein kinase p-AKT, increasing of caspase-3 and caspase-8 activities	[101]

**Table 7:** Antiprotozoal, antifungal and antiviral activities of sea cucumber bioactive compounds.

Compounds	Source	Targets	Reference
Fucosylated chondroitin sulfate	*Holothuria grisea*	*Plasmodium falciparum*	[6,103]
Fucosylated chondroitin sulfate	*Isostichopus badionotus*	*Plasmodium falciparum*	[6]
Holothurin B	*Actinopyga lecanora*	*Leishmania donovani*	[104]
Holothurin B	*Actinopyga lecanora*	*Candida albicans, Candida neoformans, Sporothrix schenckii, Trychophyton mentagrophytes, Aspergillus fumigatus*	[105]
Marmoratoside A, bivittoside D, impatienside A, 17α-hydroxy impatienside	*Bohadschia marmorata*	*Candida albicans*, *Candida neoformans*, *Sporothrix schenckii*, *Trichophyton mentagrophytes*, *Aspergillus fumigatus*, *Candida parapsilosis*	[106,107]
Holotoxins B, A, A1	*Apostichopus japonicus*	*Candida* species	[108]
Fucosylated chondroitin sulfate	*Thelenota ananas*	Human immunodeficiency virus (HIV)	[109]

## Abbreviations

ABTS	Azino-bis-3-ehtylbenzothiazoline-6-sulfonic acid
APTT	Activated partial thromboplastin time
AT III	Antithrombin III
EPA-enriched PL	Eicosapentaenoic acid-enriched phospholipids
Fuc	α-l-Fucose
FuCS	Fucosylated chondroitin sulfate
GalNAc	*N*-Acetyl-β-d-galactosamine
GlcUA	β-d-Glucuronic acid
HC II	Heparin cofactor II
HFSD	High-fat high-sucrose
MIC	Minimum inhibitory concentration
NAFLD	Non-alcoholic fatty liver disease
PPAR	Peroxisome proliferator-activated receptor
PT	Prothrombin time
SCD	Stearoyl CoA desaturase
SREBP	Sterol-regulatory element binding protein
TT	Thrombin time
